# MYB43 in Oilseed Rape (*Brassica napus*) Positively Regulates Vascular Lignification, Plant Morphology and Yield Potential but Negatively Affects Resistance to *Sclerotinia sclerotiorum*

**DOI:** 10.3390/genes11050581

**Published:** 2020-05-22

**Authors:** Jiayi Jiang, Xueli Liao, Xiaoyun Jin, Li Tan, Qifeng Lu, Chenglong Yuan, Yufei Xue, Nengwen Yin, Na Lin, Yourong Chai

**Affiliations:** 1 College of Agronomy and Biotechnology, Southwest University, Chongqing 400715, China; jjy326@sina.cn (J.J.); shrilly1002@163.com (X.L.); jxy0606@163.com (X.J.); tanli15208602588@163.com (L.T.); luiqfeng@163.com (Q.L.); 15517330669@163.com (C.Y.); swuxueyufei@163.com (Y.X.); nwyin80@126.com (N.Y.); linna123@yeah.net (N.L.); 2 Academy of Agricultural Sciences, Southwest University, Chongqing 400715, China; 3 Chongqing Engineering Research Center for Rapeseed, Southwest University, Chongqing 400715, China; 4 Chongqing Key Laboratory of Crop Quality Improvement, Southwest University, Chongqing 400715, China; 5 Engineering Research Center of South Upland Agriculture of Ministry of Education, Southwest University, Chongqing 400715, China

**Keywords:** oilseed rape (*Brassica napus*), *MYB43*, xylem, vessel, interfascicular fiber, plant morphology, lodging, yield potential, resistance, *Sclerotinia sclerotiorum*

## Abstract

*Arabidopsis thaliana* MYB43 (AtMYB43) is suggested to be involved in cell wall lignification. PtrMYB152, the *Populus* orthologue of *AtMYB43*, is a transcriptional activator of lignin biosynthesis and vessel wall deposition. In this research, *MYB43* genes from *Brassica napus* (rapeseed) and its parental species *B. rapa* and *B. oleracea* were molecularly characterized, which were dominantly expressed in stem and other vascular organs and showed responsiveness to *Sclerotinia sclerotiorum* infection. The *BnMYB43* family was silenced by RNAi, and the transgenic rapeseed lines showed retardation in growth and development with smaller organs, reduced lodging resistance, fewer silique number and lower yield potential. The thickness of the xylem layer decreased by 28%; the numbers of sclerenchymatous cells, vessels, interfascicular fibers, sieve tubes and pith cells in the whole cross section of the stem decreased by 28%, 59%, 48%, 34% and 21% in these lines, respectively. The contents of cellulose and lignin decreased by 17.49% and 16.21% respectively, while the pectin content increased by 71.92% in stems of RNAi lines. When inoculated with *S. sclerotiorum*, the lesion length was drastically decreased by 52.10% in the stems of transgenic plants compared with WT, implying great increase in disease resistance. Correspondingly, changes in the gene expression patterns of lignin biosynthesis, cellulose biosynthesis, pectin biosynthesis, cell cycle, SA- and JA-signals, and defensive pathways were in accordance with above phenotypic modifications. These results show that BnMYB43, being a growth-defense trade-off participant, positively regulates vascular lignification, plant morphology and yield potential, but negatively affects resistance to *S. sclerotiorum*. Moreover, this lignification activator influences cell biogenesis of both lignified and non-lignified tissues of the whole vascular organ.

## 1. Introduction

There are approximately 375,000 species of vascular plants globally that generate over 90% of terrestrial productivity [1]. Plant vascular tissues evolved as early as the Silurian period some 430 million years ago. The evolution of vascular tissues connected the leaves and other parts of the shoot with the roots and solved the problem of long-distance transport of water, nutrients and signaling molecules, thus enabled early vascular plants to gradually colonize the land [2,3,4]. The plant cell wall plays a dual role in the structure and function at the levels of the cell and the whole plant; it represents key determinants of cell division, differentiation and expansion, overall plant form, plant growth and development, as well as a variety of biotic and abiotic stress responses [5,6]. Among them, the plant cell wall is the physical barrier against pathogens. The majority of pathogens need to degrade the cell wall components to invade the host tissue, so the integrity of the cell wall greatly affects the sensitivity of plants to pathogens [7,8,9]. The cell walls of vascular bundles deposit a large amount of cellulose, lignin and pectin, which are closely related to the support and resistance of plants. Cellulose is the skeleton of vascular bundles and one of the main structural polymers of the cell wall, which significantly endows plants with mechanical strength [10,11], and many researchers have shown that the content of cellulose is inverse to the ability of plants to resist pathogens [10,12,13,14]. Lignin plays an important role in plant growth and development, lodging resistance and structural support. Its deposition, and the deposition of unpolymerized lignin monomers, are considered to be a physical barrier to prevent infection in the interaction between plant and pathogen, and its metabolism can also be actively involved in plant lodging resistance and in response to various environmental stresses [10,15,16]. Pectin influences the porosity of the cell wall, growth and expansion of cells, cell-to-cell adhesion and responses to pathogens [17]; its cleavage fragment oligogalacturonides (OGA) has been proven to cause a variety of defense responses and regulate growth and development in the process of interaction between plant and pathogen [18]. In summary, plant vascular tissues, cell wall or cellulose, lignin and pectin in the cell wall all not only play a crucial role in the transport of essential substances required for plant growth and development, but also provide physical structure, support and resistance/adaptability to plant bodies.

MYB transcription factors compose one of the largest family of transcription factors in plants [19]. It has been a hotspot in the study of the plant transcription factor’s function because of its large number of genes, functions and different types. Considering the significant progress in research, a large number of MYB transcription factors have been identified in all kinds of plants. The MYB transcription factor is named for its conserved DNA-binding domain (MYB domain) at its N-terminus [20,21]. The MYB domain is typically composed of 1–4 incomplete repeats (R), each R consisting of about 51–52 amino acid residues. Each R is folded into a helix–turn–helix (HTH) spatial structure that binds to the ditch of the target DNA, thereby adjusting the opening of the target gene promoter. MYB proteins can be divided into the following four main classes: 1R (R1/2, R3-MYB), 2R (R2R3-MYB), 3R (R1R2R3-MYB), and 4R (R1/R2-like repeats) depending on the number of R [20,22,23,24]. MYB43 belongs to R2R3-MYB, and most of the *MYB* genes in plants are R2R3-MYB. According to published literatures, there are 138 *R2R3-MYB* genes in the model plant *Arabidopsis thaliana*, 222 in apple (*Malus domestica*), 118 in grapevine (*Vitis vinifera*), 192 in poplar (*Populus trichocarpa*), 61 in moss (*Physcomitrella patens*), 126 in rice (*Oryza sativa*), 244 in soybean (*Glycine max*), 134 in sesame (*Sesamum indicum*), 256 in Chinese cabbage (*Brassica rapa*) and 424 in oilseed rape (*Brassica napus*) [20,25,26,27,28,29,30,31]. MYB transcription factors are involved in regulating almost all aspects of plant growth, development and metabolism during the whole of the plant’s life. They mainly regulate plant responses to biotic and abiotic stresses, cell proliferation and differentiation, histomorphogenesis, organ formation and the contents and types of primary and secondary metabolites of plant metabolic pathways [19,20,32,33,34,35,36]. Although members of the MYB superfamily have been annotated in *A. thaliana* and many other plants, most of them have not been functionally characterized.

The *R2R3-MYB* gene family in *A. thaliana* was categorized into 25 subgroups (SGs) (S1 to S25) on the basis that conserved amino-acid sequence motifs present carboxyterminal to the MYB domain, with some SGs unnamed (including the SG containing MYB43) [20,25,37]. As more and more plant genomes were sequenced and annotated, a comprehensive comparative genomic study of R2R3-MYB proteins from multiple plant species led to the identification of ten new subgroups, namely, SAt35, SAt46, SAt47, SAt59, SAt71, SAt85, SAt88, SAt91, SAt103 and SAt125, among which the subgroup SAt85 contained MYB20, MYB40, MYB42, MYB43, MYB85 and MYB99 [38,39]. In these recent phylogenetic analyses, subgroups S13, SAtM46, SAtM103, S16 and SAtM85 form an independent branch, indicating similar structural features and similar or related functional relationships among these subgroups. Actually, MYB26 from S13, MYB46 and MYB83 from SAtM46, MYB103 from SAtM103 and MYB85 from SAtM85 have been identified as positive regulators of biogenesis or deposition of the secondary cell wall by stimulating the biosynthesis of lignin, cellulose and other secondary cell wall ingredients in vascular tissues, the anther wall or pollen coat [23,40]. Geng et al. found that MYB20, MYB42, MYB43 and MYB85, which belong to the subgroup SAtM85, redundantly activate lignin biosynthesis by researching double mutants *myb20/43* and *myb42/85* and quadruple mutant *myb20/42/43/85* [41]. Zhong et al. demonstrated that *AtMYB43* is the direct target gene of AtMYB46, which is a gatekeeper involved in vascular bundle formation and secondary wall development [42,43,44]. Ehlting et al. proposed that AtMYB43 was a possible candidate transcription factor of fiber development [45]. The function of *AtMYB43* is still in the speculative stage; therefore, we do not know its systemic real function in *A. thaliana* yet. Poplar *MYB152* is a homologue gene of *AtMYB43*. After overexpressing *PtrMYB152* in poplar or *A. thaliana*, PtrMYB152 increased xylem thickness, secondary cell wall thickness, the content of lignin and expression of the genes related to lignin biosynthesis pathway in the stem of transgenic plants. These results suggest that PtrMYB152 is a specific transcriptional activator in poplar or *Arabidopsis* lignin biosynthesis [46,47]. 

*Brassica napus* (rapeseed or rape, AC genome, *n* = 19) is a recent allotetraploid species that was formed as a result of spontaneous interspecific hybridization between *B. rapa* (A genome, *n* = 10) and *B. oleracea* (C genome, *n* = 9) [48,49]. *B. napus*, which belongs to the Brassicaceae family, is an important oilseed crop globally. Its production is limited by a variety of biotic and abiotic stresses, especially for lodging and *Sclerotinia sclerotiorum* stem rot. Lodging has been shown to result in a yield reduction of as much as 46% [50], and *S. sclerotiorum* could cause 10%–80% yield loss in oilseed rape [51]. Therefore, it is very important to breed novel varieties with strong resistance to lodging and *S. sclerotiorum*. This is the core goal of contemporary oilseed rape breeding.

In this study, the *MYB43* gene families of *B. napus* and its parental species *B. rapa* and *B. oleracea* were cloned, and the *BnMYB43* gene family was silenced by RNA interference in *B. napus*. The plant morphology, yield potential, vascular lignification, xylem thickness and cell numbers were drastically inhibited, the lodging resistance was considerably weakened, but the resistance to *S. sclerotiorum* was greatly enhanced accompanied with great increase in pectin content, after suppression of the *BnMYB43* gene family in oilseed rape. Consistently, the expression of genes related to cell wall biogenesis, cell cycle and plant-pathogen interactions had the similar changes as the above results. This is consistent with the general belief that there is an antagonistic relationship between plant growth and defense [52]. It shows that *BnMYB43* is located on the node of plant growth-defense trade-offs. These results are also beneficial to contemporary oilseed rape breeding or others crops. As of now, it is not well clear about the regulatory mechanisms among vascular bundle formation, biochemical composition changes of secondary xylem, plant morphogenesis and plant disease resistance, or the occurrence and transmission of defense signals in plant growth and development. Therefore, the research based on the special regulatory node of BnMYB43 is very necessary.

## 2. Materials and Methods 

### 2.1. Plant Materials and Growth Conditions

*B. napus* cv. Zhongyou 821 (ZY821), *B. rapa* ssp. *oleifera* and *B. oleracea* var. *acephala* were used for gene cloning and expression characterization. *B. napus* cv. Zhongshuang 10 (ZS10) was used for genetic transformation. All materials were planted in growth chambers, green houses or cages of Chongqing Oilseed Rape Engineering Technology Research Center (CRTRC, Beibei, Chongqing, China (29°48′50″ N, 106°24′28″ E)) under normal agronomic procedures. For gene cloning and organ-specificity characterization, samples of the root, leaf, stem, flower and seed (ca. 30 days after flowering) of ZY821 were collected. For stem tissue-specificity characterization, the following ZY821 samples were collected: Growth cone tip, main-stem primordium, initially lignified main-stem, semi-lignified main-stem, completely lignified main-stem, semi-mature stem, mature stem, pith, xylem and bark (remaining tissues except pith and xylem in the mature stem, mainly containing phloem, cortex and epidermis). Stems of transgenic and non-transgenic control materials were also collected.

### 2.2. Fungi Strains and Growth Conditions

The strain of *S. sclerotiorum* used in this experiment was isolated and preserved by CRTRC. *S. sclerotiorum* was activated and cultured with potato media (PDA) at 22 °C with 85% humidity.

### 2.3. Nucleic Acid Isolation

Total RNA was isolated from samples using EASYspin plant RNA rapid extraction kit (Biomed, China). Total DNA was extracted from leaves using a traditional hexadecyltrimethylammonium bromide method (CTAB) [53]. The quality and quantity of nucleic acids were detected by agarose gel electrophoresis and ultraviolet spectrophotometer (NanoDrop 2000c, Thermo Fisher, Waltham, MA, USA).

### 2.4. Gene Cloning

The 5′RACE and 3′RACE-specific primers of *BnMYB43* were designed based on multiple alignments of the *MYB43* sequence of Brassicaceae. According to the SMARTer RACE Amplification Kit (Clontech, Mountain View, CA, USA), 1 µg of equally proportioned (w/w) mixture of total RNA of each organ of the roots, leaves, stems, flowers and seeds of ZS10 was used as the template to perform the reverse-transcription to synthesize first-strand total cDNA of 5′- and 3′-RACE, respectively.

The primer pairs RBnMYB43-51 + LUPM and RBnMYB43-52 + NUP (Table S1) were used for the primary amplification and the nested amplification of the 5′-RACE of the *BnMYB43* gene family, while primer pairs FBnMYB43-31 + LUPM and FBnMYB43-32 + NUP (Table S1) were used for the primary amplification and the nested amplification of the 3′-RACE of the *BnMYB43* gene family, respectively. Electrophoresis of PCR products, gel recovery, TA cloning, *Escherichia coli* transformation, colony culture and other experiments were performed using conventional methods.

A comprehensive analysis of the results of RACE cloning and in-silico cloning (*Brassica MYB43* chromosome regions, annotated mRNAs, ESTs and TSAs) indicated that there were four *MYB43* genes from *B. napus* and two *MYB43* genes from each parental species. End-to-end primer pairs FBrMYB43-1 + RBrMYB43-1, FBoMYB43-1 + RBoMYB43-1, FBrMYB43-2 + RBrMYB43-2 and FBoMYB43-2 + RBoMYB43-2 (Table S1) were designed to amplify the full-length cDNA and gDNA sequences of *BnMYB43-1/BrMYB43-1*, *BnMYB43-2/BoMYB43-1*, *BnMYB43-3/BrMYB43-2*, *BnMYB43-4/BoMYB43-2*, using mixed cDNA of various organs and total DNA of leaves of each species as templates, respectively. The GenBank accession numbers for them are as follows: *gBnMYB43-1*: MN508342; *cBnMYB43-1*: MN508343; *gBnMYB43-2*: MN508344; *cBnMYB43-2*: MN508345; *gBnMYB43-3*: MN508340; *cBnMYB43-3*: MN508341; *gBnMYB43-4*: MN508346; *cBnMYB43-4*: MN508347; *gBrMYB43-1*: MN508352; *cBrMYB43-1*: MN508353; *gBrMYB43-2*: MN508354; *cBrMYB43-2*: MN508355; *gBoMYB43-1*: MN508348; *cBoMYB43-1*: MN508349; *gBoMYB43-2*: MN508350; *cBoMYB43-2*: MN508351.

### 2.5. Quantitative RT-PCR (qRT-PCR) Analysis of Gene Expression

The total RNA of each organ/tissue sample was reverse-transcribed using PrimeScript™ 1st Strand cDNA Synthesis Kit (TaKaRa, Dalian, China) to obtain the corresponding first-strand total cDNA. All cDNA samples were diluted 30-fold in sterile water for the qRT-PCR reaction. Gene-specific primers for qRT-PCR detection of overall and member-specific expression of *B. napus*, *B. rapa* and *B. oleracea MYB43* families are listed in Table S1, and *25SrRNA* gene was detected as the internal standard. qRT-PCR analysis was performed on the CFX Connect™ Real-Time PCR Detection System (Bio-Rad, Berkeley, CA, USA) with FastStart Universal SYBR Green Master reagents (Roche, Basel, Switzerland). The program was 95 °C for 10 min, followed by 45 cycles of amplification (95 °C for 10, 58–64 °C for 30 s). After the PCR was completed, the temperature was raised from 65 to 95 °C, and the melting curve was detected to confirm the specificity of the amplification. Three replicates were performed. All data were analyzed by using CFX Manager 3.1 (Bio-Rad, Berkeley, CA, USA) with the 2^-ΔΔCT^ method.

### 2.6. Subcellular Localization

The coding sequence of *BnMYB43-1* was amplified by primer pair FBMYB43SL + RBMYB43SL (Table S1), cloned and named as *BnMYB43SL*, which was subcloned into pEGAD using *Eco*RI + *Bam*HI double-digestion, and Enhanced Green Fluorescent Protein (EGFP) was fused at the N-terminus to form subcellular localization expression vector pEGAD-*BMYB43SL* (Figure S1). The expression vector was transformed into tobacco leaves by *Agrobacterium tumefaciens*, cultured at 28 °C for two days and photographed with a confocal microscope (LSM 800, ZEISS, Jena, Germany).

### 2.7. RNAi Vector Construction

A 481-bp C-terminus coding region cDNA of *BnMYB43* family was amplified from the cDNA library using the primer pair FBnMYB43I + RBnMYB43I (Table S1), cloned and named as *BnMYB43I* (Figure S2a). This cDNA fragment had a >86.7% identity match with all *BnMYB43* genes, and no identity match was found with other sequences by BLASTing the whole *B. napus* genome (http://www.ncbi.nlm.nih.gov/genome/?term=Brassica+napus). At the 5′ end, primer FBnMYB43I contained *Bam*HI and *Aat*II restriction sites, and primer RBnMYB43I contained *Xba*I and *Nco*I restriction sites. An antisense fragment of *BnMYB43I* (*BnMYB43IA*) was subcloned into pFGC5941M using *Nco*I+*Aat*II double-digestion to generate intermediate vector pFGC5941M-*BnMYB43IA*. A sense fragment of *BnMYB43I* (*BnMYB43IS*) was subcloned into pFGC5941M-*BnMYB43IA* using *Bam*HI+*Xba*I double-digestion to generate RNAi vector pFGC5941M-*BnMYB43I* (Figure S2b). *BnMYB43I* was driven by the CaMV35S promoter. pFGC5941M-*BnMYB43I* plasmids were introduced into *A. tumefaciens* strain LBA4404 by conventional freeze–thaw transformation method to generate engineering strain.

### 2.8. Plant Transformation

*Agrobacterium tumefaciens* LBA4404 engineering strain harboring pFGC5941M-*BnMYB43I* was used to transform hypocotyl segments of *B. napus* cv. ZS10 using the method described by Cardoza and Stewart [54] with several modifications. The seeds were surface-sterilized for 1 min with 95% ethanol, sterilized for 10 min with 0.1% HgCl_2_, then were washed thoroughly with sterile distilled water, and finally germinated on MSg medium with 2.2 g/l MS (Murashige and Skoog) powder (Duchefa, Haarlem, The Nethelands) and 30 g/l sucrose, solidified with 3 g/l Gellan Gum (Zhejiang Zhongken, China), at 25 °C and 16 h light/8 h dark for 6–10 days. Hypocotyl segments of 0.5–cm length were preconditioned on MSp medium with 4.41 g/l MS, 1 mg/l 2,4-D (2,4-dichlorophenoxy acetic acid), 1 mg/l 6-BA (6-Benzylaminopurine) and 30 g/l sucrose, solidified with 3 g/l Gellan Gum, at 25 °C and 16 h light/8 h dark for two days. The LBA4404 strain was activated and subcultured to OD_600_ = 0.5 in liquid LB + Kan medium, pelleted and re-suspended in MSm medium with 4.41 g/l MS, 30 g/l sucrose, 1 mg/l 2,4-D, 1 mg/l 6-BA and 100 μmol/L AS (acetosyringone). The preconditioned hypocotyl segments were dipped in LBA4404+MSm solution for 5–10 min. After removing excess liquid with sterilized filter paper, the segments were co-cultured on MSc medium with 4.41 g/l MS, 30 g/l sucrose, 1 mg/l 2,4-D, 1 mg/l 6-BA and 100 μmol/L AS, solidified with 3 g/l Gellan Gum, at 25 °C for two days in dark. Then they were transferred to MSi medium with 4.41 g/l MS, 30 g/l sucrose, 1 mg/l 2,4-D, 1 mg/l 6-BA, 500 mg/l Cef and 30 mg/l Basta, solidified with 3 g/l Gellan Gum, at 25 °C and 16 h light/8 h dark until resistant calli were induced (ca. 2 months). The explants with calli were moved to MSd medium with 4.41 g/l MS, 30 g/l sucrose, 4 mg/l 6-BA, 2 mg/l trans-ZT, 5 mg/l AgNO_3_, 500 mg/l Cef and 30 mg/l Basta, solidified with 3 g/l Gellan Gum, at 25 °C and 16 h light/8 h dark for callus differentiation (one to several months). The differentiated calli on explants were transferred to MSs medium with 4.41 g/l MS, 30 g/l sucrose, 3 mg/l 6-BA, 2 mg/l trans-ZT, 500 mg/l Cef and 10 mg/l Basta, solidified with 3 g/l Gellan Gum, at 25 °C and 16 h light/8 h dark until the buds were formed (shooting, one to several months). The buds were transferred to MSe medium with 4.41 g/l MS, 30 g/l sucrose, 0.005 mg/l 6-BA, 500 mg/l Cef and 5 mg/l Basta, solidified with 3 g/l Gellan Gum, at 25 °C and 16 h light/8 h dark until proper shoot elongation. The elongated shoots were transferred to MSr medium with 4.41 g/l MS, 30 g/l sucrose and 0.5 mg/l NAA, solidified with 3 g/l Gellan Gum, at 25 °C and 16 h light/8 h dark to promote rooting. The regenerated plants were transplanted in pots in growth chamber and were primarily screened by dipping with 200 mg/L Basta solution on the leaves. The Basta-resistant plants were identified by PCR detection of the leaf DNA using primer pairs F35S3N + RBnPAP2I2 and FBnPAP2I2 + ROCST5N (Table S1). Subsequently, the RNA of positive transgenic plants was extracted, and the elite plants were screened out by qRT-PCR detection for subsequent analysis (Figure S3). T_2_ and T_3_ transgenic plants without segregation represent homozygous lines. Homozygous T_2_ and T_3_ transgenic plants were used for functional and mechanism analyses, and non-transgenic plants were used as control (WT).

### 2.9. Assessment of Resistance to S. sclerotiorum

The method of leaf inoculation was based on the description of Godoy et al. [55] with several modifications. The third or fourth leaf, which was numbered from the top to the bottom, was excised from each plant at the 9- to 12-leaf stage, with at least 6 leaves per line. The leaves were placed into a plastic basket with wet filter-paper. The PDA discs (7 mm in diameter) containing *S. sclerotiorum* hypha were inoculated at the center of the left and right leaf abdomens, and the ends of the petioles were wrapped with wet tissue to prevent the leaves from losing water. Finally, the basket was covered with cling film and kept at 22 °C. After 48 h of incubation, the long diameter (a) and the short diameter (b) of the lesion were measured. The lesion area was calculated as S = (π × a × b) / 4. The method of stem inoculation was based on the description by Mei et al. [56] with several modifications. The stems were inoculated at the full-bloom stage, 6 plants per line. About 45 cm-long stem fragments were cut off at 20 cm from the ground, and both ends were wrapped with cling film to prevent water loss. The PDA discs (7 mm in diameter) containing *S. sclerotiorum* hypha were inoculated at two points with an interval of 10 cm on the stem segment. Lesion lengths were measured at 96 h after inoculation.

### 2.10. Histological Analysis and Microscopic Observation

Mäule staining and Phloroglucinol-HCl staining of lignin were performed using methods of the literatures [57,58]. Cellulose staining by Fast Green FCF and pectin staining by Hydroxylamine method (Leagene, Beijing, China) were also performed. Stained sections were observed with the stereo light microscope (Olympus SZX2-FOA, Tokyo, Japan) and the fluorescence microscope (Nikon Eclipse E600W, Tokyo, Japan).

### 2.11. Determination of Cell Wall Composition

At the harvest stage, the mid-region fragments of mature stems were sampled and incubated at 70 °C to constant weight. Then, they were powdered by the universal grinder, and the powders were screened by a 100-mesh sieve. The cellulose, lignin and pectin from the cell walls of transgenic and control plants were extracted and determined. The extraction and determination of cellulose and lignin followed the method of Foster et al. [59], while extraction and quantification of pectin followed the method of Blumenkrantz and Asboe-Hansen [60]. The absorbance of cellulose, lignin and pectin was determined using an Infinite M200 Pro Microplate Reader (Tecan, Männedorf, Switzerland). There is no study on the lignin extinction coefficient of *B. napus*, therefore the value 23.35 g^−1^ L cm^−1^ at 280 nm from the model-plant and relative species *A. thaliana* [61] was used for lignin content calculation.

### 2.12. Statistical Analysis

In this research, at least three biological replications (*n* ≥ 3) were designed for each experiment. All values are means ± standard deviation (SD). Asterisks indicate significant or extremely significant differences from the control (* for 0.01 ≤ *p* < 0.05, and ** for *p* < 0.01) using one-way ANOVA.

## 3. Results

### 3.1. Cloning and Characterization of the MYB43 Gene Families from B. napus and Its Parental Species B. rapa and B. oleracea

In order to isolate *MYB43* gene family from *B. napus*, we designed conservative primers (Table S1) for 5′RACE and 3′RACE amplifications. Using end-to-end primer pairs, the full-length gDNA and cDNA sequences of *MYB43* gene family from *B. napus*, *B. rapa* and *B. oleracea* were cloned. There are four members in the *BnMYB43* gene family, namely *BnMYB43-1*, *BnMYB43-2*, *BnMYB43-3* and *BnMYB43-4*, with gene length of 2293, 2252, 2237 and 2256 bp, mRNA length of 1235, 1236, 1199 and 1217 bp, encoding polypeptides of 319, 304, 313 and 313 aa, respectively. There are two members in both the *BrMYB43* and *BoMYB43* gene family, named *BrMYB43-1*, *BrMYB43-2*, *BoMYB43-1* and *BoMYB43-2*, with gene length of 2295, 2240, 2259 and 2258 bp, mRNA length of 1235, 1202, 1242 and 1215 bp, encoding polypeptides of 319, 313, 329 and 313 aa, respectively. According to 5′RACE, 3′RACE and in-silico cloning results, *BnMYB43-1*, *BnMYB43-2*, *BnMYB43-3* and *BnMYB43-4* have a longest 5′UTR of 93, 95, 81 and 109 bp with 3 (G_1_, G_86_ and G_113_), 3 (G_1_, C_48_ and G_86_), 1 (C_1_) and 2 (A_1_ and G_99_) alternative transcription start sites, and a longest 3′UTR of 182, 226, 176 and 166 bp with 3 (G_2264_, T_2283_ and T_2293_), 3 (T_2202_, G_2236_ and G_2252_), 2 (C_2088_ and C_2237_) and 2 (T_2212_ and C_2256_) alternative poly A tailing sites, respectively. On gene level, *BnMYB43-1*, *BnMYB43-2*, *BnMYB43-3* and *BnMYB43-4* have 99.7%, 97.6%, 99.6% and 95.5% of identities to parental-species donor genes *BrMYB43-1, BoMYB43-1, BrMYB43-2* and *BoMYB43-2*, respectively; these *Brassica MYB43* genes show 71.8% to 74.0% identities to *AtMYB43* gene (Figure S4a). Like *AtMYB43*, they contain three exons and two introns (Figure S4b), and their proteins show motif patterns similar to AtMYB43. BnMYB43-1, BnMYB43-2, BnMYB43-3 and BnMYB43-4 proteins share identities/positives of 99.4%/99.4%, 94.4%/95.7%, 99.4%/99.7% and 99.4%/99.4% to BrMYB43-1, BrMYB43-2, BoMYB43-1 and BoMYB43-2, respectively; these *Brassica* MYB43 proteins show 71.9%–78.7% of identities and 76.0%–83.8% of positives to AtMYB43 (Figure S4c). Phylogenetic analysis indicated short distances among *Brassica* MYB43 proteins, and they were clustered together with AtMYB43 (Figure S4d). These results clearly demonstrate that all the *Brassica MYB43* genes isolated here are orthologues of *AtMYB43*.

### 3.2. The BnMYB43 Gene Family was Dominantly Expressed in the Xylem of the Mature Stem

**Figure 1:**
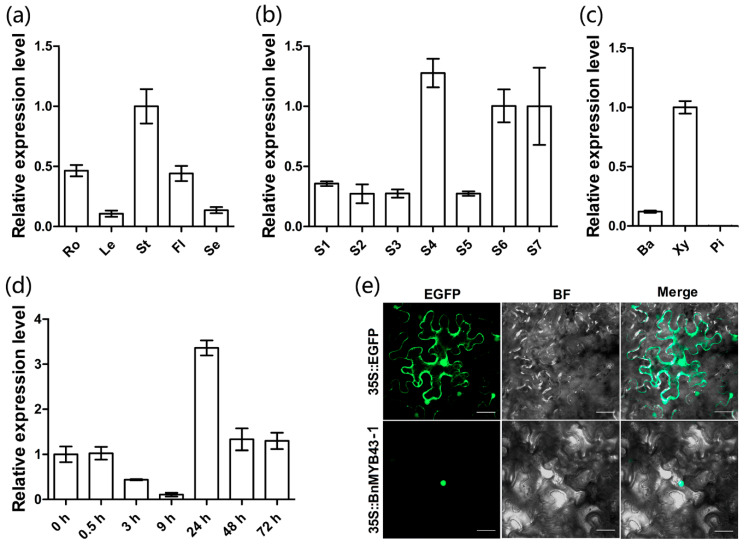
Expression pattern of *BnMYB43* gene family in oilseed rape and subcellular localization of BnMYB43-1 in *Nicotiana benthamiana*. (**a**) Expression analysis of *BnMYB43* gene family in organs of *B. napus.* (**b**) Expression analysis of *BnMYB43* gene family in stems of *B. napus*. (**c**) Expression analysis of *BnMYB43* gene family in stem tissues of *B. napus*. (**d**) Expression analysis of *BnMYB43* gene family after inoculation by *S. sclerotiorum*. (**e**) Subcellular localization of BnMYB43-1 in the nucleus. The expression levels were relative to stem (**a**), S7 (**b**), Xy (**c**) and 0 h (**d**), which were set to 1. Expression level of *25SrRNA* was used as a control reference gene. Ro, root; Le, leaf; St, stem; Fl, flower; Se, seed (30 DAP); S1, growth cone tip; S2, main-stem primordium; S3, initially lignified main-stem; S4, semi-lignified main-stem; S5, completely lignified main-stem; S6, semi-mature stem; S7, mature stem; Ba, bark; Xy, xylem; Pi, pith; Bars, 50 μm (**e**). Values are means ± SD (standard deviation) from three biologically independent repeats.

According to qRT-PCR results, *BnMYB43* overall expression could be detected in all organs of *B. napus* but varied greatly among organs; it was highest in stem, moderate in the root and flower, low in the leaf and lowest in the seed (Figure 1a). It is clear that the *BnMYB43* gene family is generally dominantly expressed in vascular organs such as stem and root. Knowing that it is dominantly expressed in vascular organ stem, we further detected its tissue-specificity. The main stem of oilseed rape ZY821 at early bolting stage (ca. 5–6 cm in height) was divided into 7 developmental segments: growth cone tip, main-stem primordium, initially lignified main-stem, semi-lignified main-stem, completely lignified main-stem, semi-mature stem and mature stem. The qRT-PCR result shows that *BnMYB43* is expressed in all parts along the main stem regardless of vascularization degree but is significantly higher in the vascularized stem than in the non-vascularized stem (Figure 1b). Furthermore, the mature stem was dissected into pith, xylem and bark for qRT-PCR detection, which indicated that *BnMYB43* gene family expression is dominant in xylem, distinct in bark and non-existent in pith (Figure 1c). This implies that the *BnMYB43* gene family may be mainly involved in vascular bundle formation or xylem development.

### 3.3. BnMYB43 Can Be Quickly Induced by S. sclerotiorum Infection

In order to confirm the *BnMYB43* response to *S. sclerotiorum* infection in oilseed rape, *S sclerotiorum* was inoculated on oilseed rape leaf. After *S. sclerotiorum* inoculation, *BnMYB43* expression was downregulated at 3 to 9 h, distinctly upregulated at 24 h, and returned to a slightly higher than basal level at 48 h, showing a dynamic as if influenced by struggling between *B. napus* and *S. sclerotiorum* (Figure 1d). This implies that the *BnMYB43* gene family may be involved in the process of interaction with *S. sclerotiorum* in oilseed rape.

### 3.4. BnMYB43 is Localized to the Nucleus

In consistence with the bioinformatics prediction and its predicted role as a transcription factor, Enhanced Green Fluorescent Protein (EGFP)-tagged BnMYB43-1 was shown to be localized in the nucleus when expressed in tobacco leaf (Figure 1e).

### 3.5. The Silencing of BnMYB43 Changed the Plant Morphology, Physiology and Yield Factors

**Figure 2:**
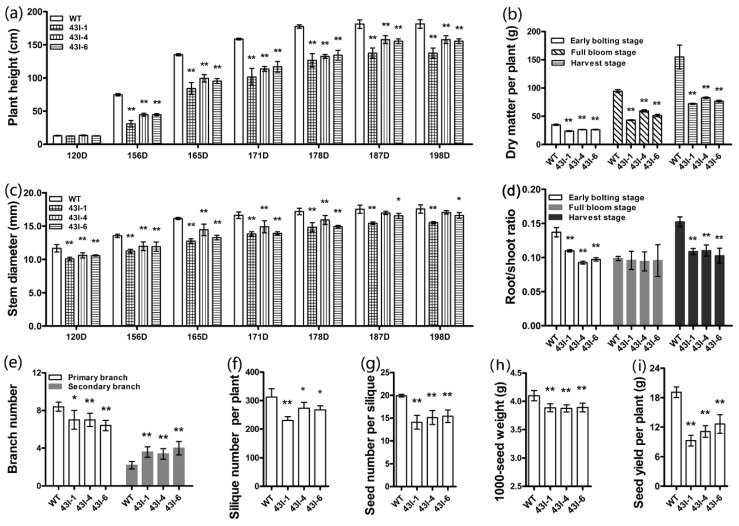
Phenotypic changes of *BnMYB43I* transgenic plants. (**a**) The development dynamic of the plant height. (**b**) The dry matter of plant. (**c**) The development dynamic of the main stem diameter. (**d**) The root/shoot radio. (**e**) Number of branches. (**f**) Number of silique at the mature stage (191D). (**g**) Number of seed per silique. (**h**) The weight of 1000-grain of oilseed rape. (**i**) The weight of the seed yield per plant. Values are means ± SD from three biologically independent repeats. Asterisks indicate significant or extremely significant differences from the control (*, 0.01 ≤ *p* < 0.05; **, *p* < 0.01) using one-way ANOVA.

After PCR genotyping (Figure S3), three elite transgenic lines of independent transformation events with *BnMYB43* RNA interference were used for subsequent investigation. Compared with non-transgenic control plants (WT), the transgenic plants were smaller in morphology, the root system and the leaf (Figure S5), the stem diameter was reduced by 6% to 16% along development stages, and the number of primary branches decreased by 19.05% (Figure 2c); however, the number of secondary branches increased by 66.67% (Figure 2e). The plant height of transgenic plants showed little difference at seedling stage, but from bolting stage to harvest stage it became 17% to 46% shorter than WT (Figure 2a). The tested dry matter of transgenic plants at early bolting stage, full bloom stage and harvest stage decreased by 26.86%, 45.69% and 50.22%, respectively (Figure 2b). The root/shoot ratio of transgenic plants at early bolting stage, full bloom stage and harvest stage decreased by 27.17%, 3.61% and 29.52%, respectively (Figure 2d). Corresponding to growth impairment, yield factors of transgenic plants were also significantly influenced, with 17.50% decrease in silique number per plant, 25.29% decrease in seed number per silique and 5.29% decrease in 1000-seed weight, and 42.43% decrease in seed yield per plant (Figure 2f–i; Figure S5g).

### 3.6. The Silencing of BnMYB43 Impaired Xylem, Especially Interfascicular Fiber Biogenesis and Stem Strength

**Figure 3:**
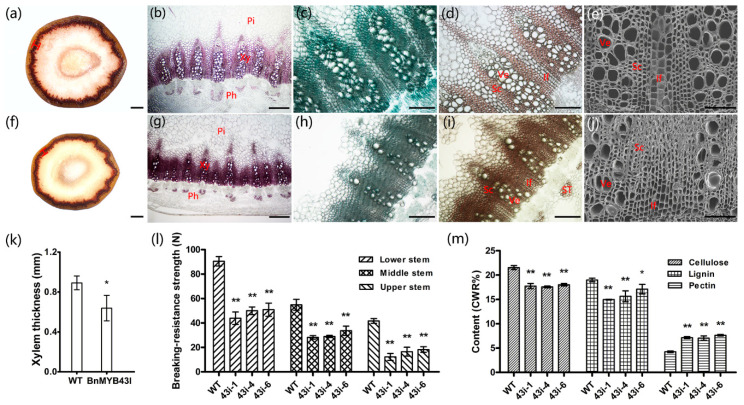
The phenotypes of morphologies, ingredients and properties of the stem in the oilseed rape at mature stage. (**a**–**e**) The cross sections of the stem in WT at mature stage. (**f**–**j**) The cross sections of the stem in the *BnMYB43I* lines at mature stage. (**a**,**f**) Mäule staining of lignin in whole cross sections. (**b**,**g**) Phloroglucinol-HCl staining of lignin in partial cross sections. (**c**,**h**) Fast Green FCF staining of cellulose in partial cross sections. (**d**,**i**) Hydroxylammonium staining of pectin in partial cross sections. (**e**,**j**) The morphologies of vessel and interfascicular fibers. (**k**) The thickness of xylem. (**l**) The breaking-resistance strength of stem. (**m**) Determination of the content of cellulose, lignin and pectin. Lengths of the I-line in (**a**) and (**f**) are the lengths of each vascular bundle, and the average of them is the thickness of xylem. Pi, pith; Xy, xylem; Ph, phloem; Ve, vessel; Sc, sclerenchymatous cell; If, interfascicular fibers; ST, sieve tube; Bar, 2 mm (**a**,**j**), 500 μm (**b**,**g**), 250 μm (**c**–**d**, **h**–**i**), 100 μm (**e**,**j**). Values are means ± SD from nine biologically independent repeats. Asterisks indicate significant or extremely significant differences from the control (*, 0.01 ≤ *p* < 0.05; **, *p* < 0.01) using one-way ANOVA.

*BnMYB43* is dominantly expressed in vascular organs, especially in xylem, and the *BnMYB43*-RNAi plants mainly showed phenotypes associated with retardation of vascular organs, therefore anatomical and histochemical microscopic observations were carried out on the middle part of the stem at harvest stage. The areas of pith and xylem in the transgenic plants were decreased compared with WT plants. The number of vascular bundles did not change significantly, while the single vascular bundle became smaller and the xylem layer became thinner in the transgenic plants than in WT plants (Figure 3a,f,k). Stained areas of lignin, cellulose and pectin were all significantly reduced, but the staining was deeper in the transgenic plants than in WT plants (Figure 3b–d, g–i). Statistical analysis showed that the cell number of pith in the whole cross section of stem in the transgenic plants decreased significantly by 21%, although there was no significant change in cell size and cell number per unit area (Table 1). The thickness of the xylem layer decreased by 28% (Figure 3a,f,k), the numbers of sclerenchymatous cell and vessel in the whole cross section of stem decreased by 28% and 59%, and the numbers of sclerenchymatous cell and vessel in a single vascular bundle of stem decreased by 24% and 56%, respectively, in the transgenic plants (Figure 3b–e,g–j; Table 1). More significantly, the number of interfascicular fibers in the whole cross section of stem in the transgenic plants decreased by 48%, and the number of interfascicular fibers between two vascular bundles of stem in the transgenic plants decreased by 45% (Table 1). In addition, the number of sieve tubes in the whole cross section and per vascular bundle of stem in the transgenic plants decreased by 34% and 30%, respectively (Table 1). An unexpected finding was that under microscope observation, the cell wall thickness of all lignified and non-lignified cells of the whole section did not show a reduction, and perhaps showed a small increase, which we can be speculated due to the somewhat denser histochemical staining of the transgenic plants. The results indicate that the silencing of *BnMYB43* mainly decreases transverse diameters of vascular organs solely by reducing cell numbers, not by reducing cell size and cell wall thickness.

Subsequently, we adopted a plant stem strength tester to test the effect of above tissue changes on stem strength, which is crucial for plant morphology and lodging resistance in cultivation [62]. The results showed that the breaking-resistance strength of the upper, middle and lower stems of transgenic plants was decreased by 62.30%, 44.71% and 46.69%, respectively, with an average value of 49.59% for the whole stem (Figure 3l), implying a decrease in lodging resistance.

### 3.7. The Silencing of BnMYB43 Profoundly Remolded Cell Wall Ingredients

To investigate the biochemical basis underlying the above-mentioned phenotypic and anatomical changes, the cellulose, lignin and pectin of stems at the harvest stage were extracted and quantified by spectrophotometry. In stems of transgenic plants, the contents of cellulose and lignin decreased significantly by 17.49% and 16.21%, respectively, while the pectin content increased significantly by 71.92% when compared with WT (Figure 3m; Table S2). In addition, the lignin monomers of the same samples were extracted and determined, showing a 19.18% reduction in lignin total content and a decrease in H-, G- and S-type lignin content by 31.64%, 31.86% and 11.21% respectively. However, changes of the proportions among the three lignin types were subtle. As H-lignin proportion decreased by 0.19 percent points (1.15% vs. 1.34%) and G-lignin proportion decreased by 5.60 percent points (31.54% vs. 37.14%), the S-lignin proportion was increased by 5.79 percent points (67.31% vs. 61.52%), and the S/G ratio was increased by 0.48 (2.14 vs. 1.66), implying that inhibition of G- and H-lignin biosynthesis was stronger than inhibition of S-lignin biosynthesis (Table S3). These results indicate that the silencing of *BnMYB43* significantly reduced deposition of secondary cell wall ingredients such as lignin and cellulose, but greatly increased the content of cell-surface wall ingredient pectin (only existing in primary cell wall), and lignin reduction was stronger in G- and H-types than in S-type.

### 3.8. The Silencing of BnMYB43 Enhanced Resistance to S. sclerotiorum Stem Rot

**Figure 4:**
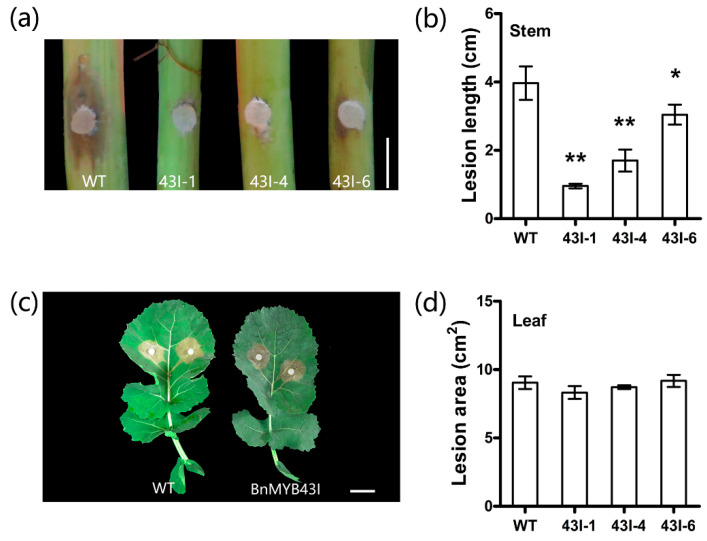
Phenotypic and lesion size of detached stems and leaves after inoculation with *S. sclerotiorum*. (**a**,**c**) The phenotypes of detached stems and leaves after inoculation with *S. sclerotiorum*, respectively. (**b**) The length of lesions in stem after inoculation with *S. sclerotiorum* for 96 h. (**d**) The area of lesions in leaf after inoculation with *S. sclerotiorum* for 48 h. Bars, 15 mm (**a**) and 30 mm (**c**). Values are means ± SD (*n* = 3 biological replicates). Asterisks indicate significant differences from the control (*, 0.01 ≤ *p* < 0.05; **, *p* < 0.01) using one-way ANOVA.

As *BnMYB43* expression is responsive to *S. sclerotiorum* infection as described above (Figure 1d), *BnMYB43*-silenced plants were identified for *S. sclerotiorum* resistance. In stem-inoculation identification, lesion length was drastically decreased by 52.10% in the transgenic plants compared to WT, implying a great increase in resistance to *S. sclerotiorum* stem rot disease (Figure 4a,b). Leaf-inoculation identification results showed a non-significant enhancement in resistance to *S. sclerotiorum* in transgenic plants (Figure 4c,d). It is clear that the suppression of *BnMYB43* greatly but solely enhanced the resistance to *S. sclerotiorum* in vascular organs in which innate *BnMYB43* is dominantly expressed.

### 3.9. The Silencing of BnMYB43 Affected Gene Expression Related to Cell Wall Biogenesis, Cell Cycle and Plant-Pathogen Interactions

**Figure 5:**
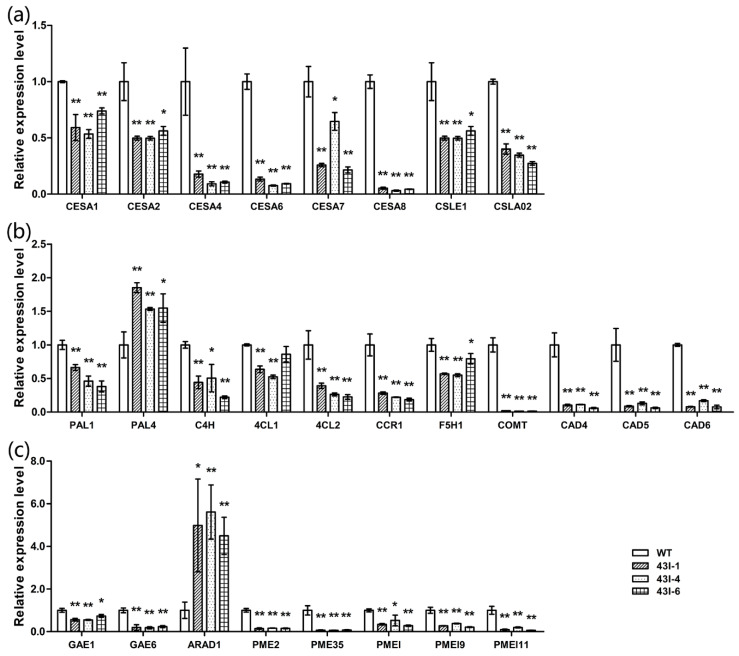
The expression patterns of genes related to cellulose (**a**), lignin (**b**) and pectin (**c**) in the stem of oilseed rape after silencing *BnMYB43* gene family. Expression level of each gene in WT was set as 1 (**a**–**c**). Expression level of *25SrRNA* was used as control reference. Values are means ± SD (*n* = 3 biological replicates). Asterisks indicate significant or extremely significant differences from the control (*, 0.01 ≤ *p* < 0.05; **, *p* < 0.01) using one-way ANOVA.

**Figure 6:**
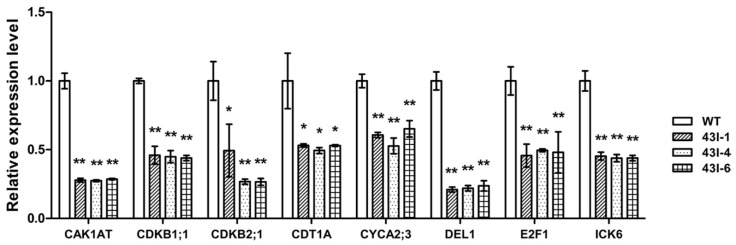
The expression patterns of genes related to cell cycle in the stem of oilseed rape after silencing *BnMYB43* gene family. Expression level of each gene in WT was set as 1. Expression level of *25SrRNA* was used as control reference. Values are means ± SD (*n* = 3 biological replicates). Asterisks indicate significant or extremely significant differences from the control (*, 0.01 ≤ *p* < 0.05; **, *p* < 0.01) using one-way ANOVA.

In order to understand the relationship between gene expression differences and phenotypic changes, the upper part of stems was sampled for qRT-PCR at the end of bolting stage. In transgenic plants, the genes *CESA1*, *CESA2*, *CESA4*, *CESA6*, *CESA7*, *CESA8*, *CSLE1* and *CSLA02*, which are related to cellulose biosynthesis, were significantly down-regulated (Figure 5a); lignin biosynthesis related genes *PAL1*, *PAL4*, *C4H*, *4CL1*, *4CL2*, *CCR1*, *F5H1*, *COMT*, *CAD4*, *CAD5* and *CAD6* were down-regulated (Figure 5b); pectin biosynthesis related genes *GAE1* and *GAE6* were significantly down-regulated, but *ARAD1* was significantly up-regulated. Additionally, the pectin methylesterase genes *PME2* and *PME35*, and pectin methylesterase inhibitor genes *PMEI*, *PMEI9* and *PMEI11*, were significantly down-regulated (Figure 5c); the cell cycle related genes *CAK1AT*, *CDKB1;1*, *CDKB2;1*, *CDT1A*, *CYCA2;3*, *DEL1*, *E2F1* and *ICK6* were also significantly down-regulated (Figure 6). These results are consistent with the results of stem cell wall ingredients (Figure 3m), the determination of stem-breaking resistance (Figure 3l), and the statistical results of the number of cells (Table 1) in the stems of transgenic plants.

**Figure 7:**
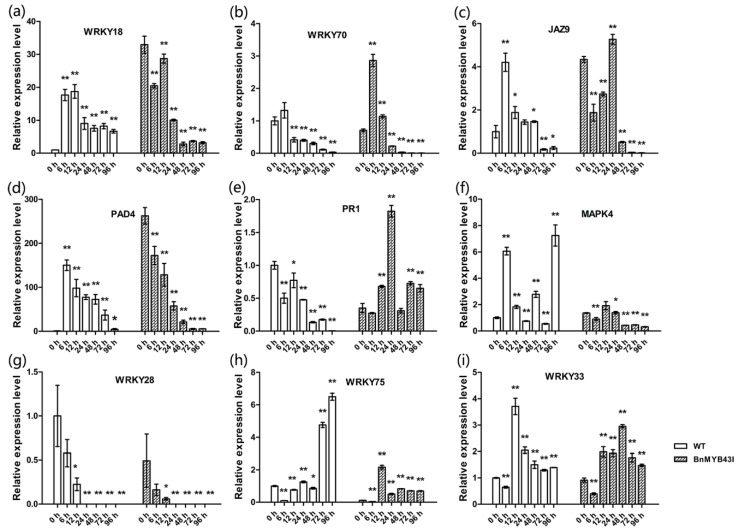
The expression patterns of genes related to plant–pathogen interactions in the plants after silencing *BnMYB43* gene family. (**a**–**c**) The expression of genes related to salicylic acid or jasmonic acid signal pathways. (**d**,**e**) The expression of other genes related to plant defense. (**f**–**i**) The expression of genes related to *S. sclerotiorum* susceptibility or resistance in *B. napus*. Expression level of each gene in WT was set as 1. Expression level of *25SrRNA* was used as control reference. Values are means ± SD (*n* = 3 biological replicates). Asterisks indicate significant or extremely significant differences from the control (*, 0.01 ≤ *p* < 0.05; **, *p* < 0.01) using one-way ANOVA.

In addition, the stems were sampled and analyzed by qRT-PCR at different times after inoculation with *S. sclerotiorum*. First, there were changes in expression of known genes of SA- or JA-mediated defense pathways. SA-pathway gene *WRKY18* was sharply up-regulated by inoculation in WT, but before inoculation its expression in transgenic plants was already up-regulated by 32 folds compared to WT. Though it showed down-regulation in transgenic plants during infection, its expression level was higher than in WT for the first 24 h (Figure 7a). The expression of *WRKY70* and *JAZ9* in transgenic plants was higher than in WT during the whole infection process, and was highly expressed at the early stage of infection (Figure 7b,c). *WRKY70* was associated with both SA- and JA-pathways and was not distinctly influenced by *BnMYB43* silencing before inoculation, while JAZ9 was associated with JA-pathway and was distinctly upregulated by *BnMYB43* silencing before inoculation. There were some changes in other known defense genes. *PAD4* was significantly increased by 262 folds before inoculation in transgenic plants compared with WT plants. In WT, *PAD4* expression was sharply up-regulated by 150-fold at 6 h after inoculation. Both transgenic and WT plants showed a down-regulation of *PAD4* along the infection process, but the expression was still higher in transgenic plants than in WT (Figure 7d). The expression of *PR1* showed an up-regulation trend during infection in transgenic plants but a down-regulation trend in WT plants along the infection process (Figure 7e). There were also expression changes in known genes of *S. sclerotiorum* resistance or susceptibility. During infection, the expression of *MAPK4* was decreased in transgenic plants while increased in WT plants, *WRKY28* showed decrease in both transgenic and WT plants, while *WRKY75* and *WRKY33* showed increase in both transgenic and WT plants (Figure 7f–i). These results showed that the reason for the increased resistance to *S. sclerotiorum* in *BnMYB43I* plants was caused by multiple related effects brought by *BnMYB43* silencing, and different defensive mechanisms responded differentially to *BnMYB43* silencing.

## 4. Discussion

### 4.1. BnMYB43 is a Pivotal Regulator of the Biogenesis of Lignified Cells in Vascular Organs

The xylem, which is a long-distance transport system, belongs to vascular tissues [63]. The xylem is composed of conductive tracheary elements and non-conductive elements. The conducting tracheary elements are vessels in angiosperms or tracheids in gymnosperms. The non-conductive elements are xylary parenchyma cells and xylary fibers [64]. The xylem can transport and store water, nutrients and hormones, provide mechanical support for the plant body, and play a very important role in the growth and development of plants. Most of the water transport is provided by vessel cells and most of the mechanical support is provided by fiber cells in angiosperms [65]. The interfascicular fibers are composed of three to four layers of fiber cells, which are mainly responsible for the mechanical strength of mature stems [66,67]. Therefore, the size and number of vessels and fiber cells are important parameters for the study of long-distance water transport, plant mechanical support, xylem adaptations or pathology of the xylem [68]. 

In this study, *BnMYB43* was dominantly expressed in the xylem of mature stems (Figure 1); then, the xylem of the stem became thinner by silencing the expression of the *MYB43* gene family in *B. napus* (Figure 3a,f,k), and the number of vessels, interfascicular fibers and other sclerenchyma cells decreased significantly in the stem (Figure 3b–e, g–j; Table 1). Innate *BnMYB43* was also expressed with certain level in the bark, and its silencing also decreased phloem cell numbers (Table 1). We determined the biochemical components of the stem cell wall of transgenic *BnMYB43*-silenced plants. The results showed that the content of cellulose and lignin decreased significantly, while the content of pectin increased significantly (Figure 3m). Consistently, cellulose and lignin biosynthesis related genes were down-regulated, pectin biosynthesis related genes were up-regulated and several pectin methylesterase and pectin methylesterase inhibitor genes were down-regulated (Figure 5). Lignin only occurs in secondary cell wall, pectin is present solely in primary cell wall, and cellulose participates in both. Decrease in lignin and cellulose and increase in pectin imply decreased lignification. These results are consistent with the results of microscopic observation of the stems (Figure 3a–d,f–i,k). Moreover, innate *BnMYB43* showed considerable expression in root and other vascular organs (e.g., leaf vein net), and *BnMYB43*-silenced plants also had smaller organs in root system and other vascular organs (e.g., leaf vein net) with short root length and less lateral root number, indicating similar effect in root other vascular organs as observed in stem. It can be inferred that *Brassica MYB43* genes are key regulatory factors of the biogenesis of lignified cells in vascular organs, stem being the major organ.

### 4.2. BnMYB43′s Function Has Effect on the Biogenesis of Parenchyma Cells

Unexpectedly, though innate *BnMYB43* expression could not be detected in the pith, the silencing plants showed a smaller pith area. The number of total pith cells in the cross section of the transgenic stem decreased significantly, although there was no significant change in the cell number per unit area (Table 1). The impairment of stem and plant development in silencing plants was caused by the reduction in cell numbers of all lignified and non-lignified tissues other than the reduced lignification degree of the sclerenchyma cells. The biogenesis of both sclerenchyma and parenchyma cells was retarded, which profoundly influenced the development of the stem and the whole plant. As for why silencing sclerenchyma-related *BnMYB43* could impair parenchyma cell biogenesis, the possible reason is signal crosstalk between these two types of tissues during stem development, which might involve cell cycle/division process.

Higher plants produce various tissues and organs of mature plants through the combination of cell division, cell expansion and cell differentiation [69,70]. Therefore, the growth and development of plants are closely related to cell division [71]. Plant cell division has a complex regulatory mechanism, and there are many molecules involved, such as CDKs, cyclins, CKS, KRPs, ICKs, CAKs, Retinoblastoma Protein and E2Fs. Among them, CDKs play a key role [69,71,72,73]. In this study, the expression of cell cycle related genes was consistently significantly down-regulated in *BnMYB43*-silencing plants when compared with WT (Figure 6), implying lower activity of cell division process in the whole organ. BnMYB43 as a key positive regulator of vascular lignification also affects the cell biogenesis of the whole organ possibly through crosstalk in cell cycle pathway.

### 4.3. BnMYB43 Participates in the Trade-Offs Between Organ Development and Disease Resistance

In this study, though plant development and growth were retarded by *BnMYB43* silencing, inoculation results indicated great enhancement in stem resistance to *S. sclerotiorum* infection (Figure 4). The first reason might be the modification of cell wall composition and structure. Due to the surface position, plant cell wall plays an important role in the interaction between the plant and microorganisms [74]. First of all, the cell wall is the natural physical barrier against the pathogen. The polysaccharides in the cell wall are crosslinked into a network through ions and covalent bonds to resist physical penetration. Secondly, the cell wall is the dynamic storage of antibacterial proteins and secondary metabolites that inhibit the growth of pathogens [75]. Studies have shown that the cellulose content in the cell wall is negatively correlated with plant disease resistance [3,10,12,14]. In other research, the content of lignin in the cell wall is proportional to plant disease resistance [14,76,77,78]. Finally, the pectin content in the cell wall is proportional to plant disease resistance [17,18]. In this study, *BnMYB43* silencing plants showed drastic reduction in cellulose and lignin contents but great increase in pectin contents in the stem, which were in consistent with changes in gene expression patterns of respective biosynthesis pathways (Figure 3 and Figure 5). It is reasonable to assume that changes in these ingredients as well as stem structure in transgenic rapeseed could influence the resistance to *S. sclerotiorum*, especially the increased pectin content and shifted methylation pattern might contribute to resistance enhancement.

The second reason, which might be more important, is modification of classical defensive genes. Contrary to the down-regulation of gene expression in lignin and cellulose biosynthesis pathways, many of the genes involved in disease resistance were up-regulated by *BnMYB43* silencing. Before inoculation, the basal expression levels of *WRKY18*, *JAZ9* and *PAD4* were 32.95, 4.34 and 262.57 folds higher in transgenic plants than in WT plants respectively, implying enhanced preexisting innate immunity. After inoculation with *S. sclerotiorum*, the expression levels of *WRKY18*, *WRKY70*, *JAZ9*, *PAD4*, *PR1* and *WRKY33* were generally higher in transgenic plants than in WT plants, implying elevated ability in combating pathogen infection process (Figure 7). On the other hand, negative factors of necrotrophic disease resistance such as *MPK4*, *WRKY28* and *KRKY75* showed lower expression levels in transgenic plants than in WT plants before or after inoculation. *WRKY18* is involved in the SA-mediated defense signaling pathway against pathogens by inducing systemic acquired resistance (SAR) [79,80]. *WRKY70* acts as an activator of SA-induced genes and a repressor of JA-responsive genes, and as a node of convergence for integrating SA- and JA-signaling events during plant defense [81,82]. *JAZ9* is a repressor that regulates JA signaling [83,84]. *AtPAD4* is an important regulator of plant defense signaling, and *PR1* is a defense-related gene [85,86,87]. *WRKY33* was proved to markedly enhance resistance to *S. sclerotiorum* in oilseed rape [88]. *MPK4* is generally regarded as a negative regulator of defense responses [89,90,91], although Wang et al. and Zhang et al. proved that overexpression of *BnMPK4* enhanced resistance to *S. sclerotiorum* and *Pseudomonas syringae* pv *tomato (Pst)* DC3000 in oilseed rape. *WRKY28* and *WRKY75* positively regulate *A. thaliana* defense against *S. sclerotiorum* [92], but negatively regulate *B. napus* defense against *Botrytis cinerea* and *S. sclerotiorum* [93,94].

These results indicate that cell wall lignification/vascular development and disease resistance have the status of growth-defense trade-offs in *BnMYB43I*-transgenic plants. The disease resistance not only comes from cell wall modification, but also be contributed by activating the expression of signal pathway and defensive genes after silencing the *BnMYB43* gene family. For *BnMYB43*, adequate expression level is crucial to maintain an optimum trade-off between growth and resistance, otherwise strengthening of one side will sacrifice the other side.

### 4.4. Involvement of BnMYB43 in Contributing to Plant Morphology and Yield Potential

The formation of lateral roots is the main determinant of root structure and an important means of effectively absorbing water and nutrients [95]. In this study, the roots of transgenic lines were less developed than WT (Figure S5a–d), so we speculate that the transgenic plants are not able to absorb as much water and nutrients for plant growth and development as the WT plants. Certainly, less developed roots were also less powerful in terms of plant anchoring in the soil in transgenic plants than WT. The leaves of the transgenic lines became shorter (Figure S5e), and the corresponding leaf area was smaller than WT (Figure S5f); therefore, the photosynthesis and transpiration of transgenic plants are likely weaker, which is not conducive to the transport of water and nutrients in the whole plant. Stems with decreases in diameter (Figure 2c), xylem thickness (Figure 3k), number of vessels and all kinds of cells (Figure 3a–j; Table 1) are also likely less not conducive to the transport of nutrients and water for the growth of transgenic plants. Consequently, the silique number and seed yield of transgenic plants decreased significantly (Figure 2f,i). The above results showed that suppression the expression of *BnMYB43* gene family affected the growth and development of root, leaf, stem, silique and seed of oilseed rape, and especially the yield. Therefore, *MYB43* is speculated to be involved in the contribution of plant morphogenesis and yield potential.

## 5. Conclusions

In this study, suppression of the expression of the *BnMYB43* gene family was shown to inhibit the growth and development of oilseed rape, reduce its yield and weaken its lodging resistance while increasing its resistance to *S. sclerotinia*. The results suggest that *BnMYB43* plays a role in the balance between growth and defense; this phenomenon is consistent with growth–defense trade-offs. In order to survive and reproduce, plants, which grow in dynamic environments where there are many threats and opportunities, have to balance their investment in growth and defense with limited energy and resources, which has been called growth–defense trade-off [96]. The growth-defense trade-off has important ecological, agricultural and economic consequences [97]. That means *BnMYB43* is an important factor in the growth-defense trade-offs of oilseed rape. The information obtained from this study can be used to guide the genetic modification of *BnMYB43*, and makes it a new breeding target for modifying the trade-off status in favor of growth, or resistance, or both.

## Figures and Tables

**Table 1:** Number of cells of pith, sclerenchyma, vessels, interfascicular fibers and sieve tubes of stems of *BnMYB43I* transgenic plants.

Genotype	Pith Cells		Sclerenchyma Cells		Vessels		Interfascicular Fibers		Sieve Tubes	
	Cross Section	Unit Area	Cross Section	Vascular Bundle	Cross Section	Vascular Bundle	Cross Section	Vascular Bundle	Cross Section	Vascular Bundle
WT	7695.48 ± 664.97	74.15 ± 4.95	74279.47 ± 4344.94	516.93 ± 21.64	6090.73 ± 595.54	42.33 ± 2.64	5137.67 ± 791.03	35.67 ± 4.36	3477.65 ± 335.67	24.21 ± 1.62
*BnMYB43I*	6083.83 ± 676.89 *	73.25 ± 5.17	53039.80 ± 8160.15 *	389.47 ± 49.32 *	2490.80 ± 395.41 **	18.27± 2.14 **	2670.73 ± 337.29 **	19.60 ± 1.64 **	2288.52 ± 251.67 **	16.83 ± 1.72 **

WT: wild type. Values are means ± SD from nine biologically independent repeats. Asterisks indicate significant or extremely significant differences from the control (*, 0.01 ≤ *p* < 0.05; **, *p* < 0.01) using one-way ANOVA.

## Supplementary Materials

The following are available online at https://www.mdpi.com/2073-4425/11/5/581/s1. Figure S1. Construction of pEGAD-*BnMYB43-1*. Figure S2. Construction of pFGC5941M-*BnMYB43I*. Figure S3. Identification of *MYB43I* transgenic lines and comparison of *BnMYB43* expression level between WT and *BnMYB43I* transgenic plants. Figure S4. Sequence analysis of the *BnMYB43* gene family from *B. napus*. Figure S5. The plant phenotype of *BnMYB43I* transgenic plants. Table S1. Primers used in this study. Table S2. Cellulose, lignin and pectin compositions measurement of the *BnMYB43I* transgenic plants. Table S3. Lignin monomer compositions measurement of the *BnMYB43I* transgenic plants.
