# Supplementary material for: MYB43 in Oilseed Rape (Brassica napus) Positively Regulates Vascular Lignification, Plant Morphology and Yield Potential but Negatively Affects Resistance to Sclerotinia sclerotiorum
Source: Genes (Basel). 2020 May 22;11(5):581. doi: 10.3390/genes11050581 (PMC7290928; doi:10.3390/genes11050581)
Supplement: Supplementary file 1 [file genes-11-00581-s001.zip › genes-794201-suppl-1/genes-794201-supplementary Figures-Revision.docx]

**
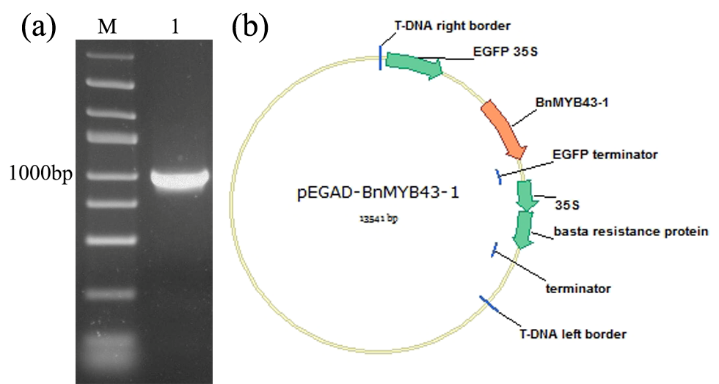
**

**Figure S1.** Construction of pEGAD-*BnMYB43-1*. (a) Amplification of *BnMYB43-1*. (b) Structure of pEGAD-*BnMYB43-1*. M: DNA marker. 1: Amplified *BnMYB43-1* band.

**
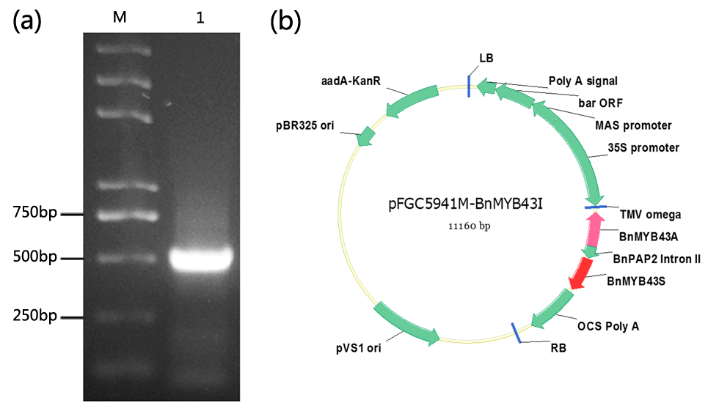
**

**Figure S2.** Construction of pFGC5941M-*BnMYB43I*. (a) Amplification of BnMYB43I. (b) Structure of pFGC5941M-*BnMYB43I*. M: DNA marker. 1: Amplified *BnMYB43I* band.

**

**

**Figure S3.** Identification of MYB43I transgenic lines and comparison of BnMYB43 expression level between WT and *BnMYB43I* transgenic plants. (a) PCR-based identification of transgenic plants using the primers given in Tables S1. Upper panel, F35S3N and RBnPAP2I2 primer pair; Lower panel, FBnMYB43I and ROCST5N primer pair. WT and pFGC5941M-*BnMYB43I* plasmid were used as negative and positive control, respectively. 1, 2 and 3 were the 43I-1, 43I-4 and 43I-6, respectively. (b) qRT-PCR results comparing *BnMYB43* expression levels in developing lateral branch between WT and MYB43I transgenic lines using *25SrRNA* as the internal reference. The expression levels of genes are relative to WT, which is set to 1. M: DNA marker; CK+: pFGC5941M-*BMYB43I* plasmid. Values are means ± SD from three biologically independent repeats. Asterisks indicate significant differences from the control (*, 0.01 ≤ P < 0.05; **, P < 0.01) using one-way ANOVA.

**(a)**

1 100

AtMYB43 gene (1) ATAGGTTGTTGTGTAGCCTCTTTAGTCTTGGAAATACAACATATTATAAGTATATTAGGTAACATATATGATCATATCTCTATATATAAAGTAGGTATAT

BnMYB43-1 gene (1) -----------------------------GGAAATACAACAT--TGTCAGTATATTAGATATC-TATTTGAGCACATCTCTAGACTATATAAAGTAAGAA

BnMYB43-2 gene (1) -----------------------------GGAAATACAACAT--TGTCAGTATATTAGATATC-TATTCGAGCACATCTCTAGACTATATAAAGAAAGAA

BnMYB43-3 gene (1) -------------------------------------------------------------CT-TGTTTGAGCATATCTCTAGACTA-ATATATATATAT

BnMYB43-4 gene (1) ---------------------------ATATACATTATAACT--TGTAAGTATATTAGGTACT-TGTTTGAGCATATCTCTAGACTA-ATAGATATATAT

BrMYB43-1 gene (1) -----------------------------GGAAATACAACAT--TGTCAGTATATTAGATATC-TATTTGAGCACATCTCTAGACTATATAAAGTAAGAA

BrMYB43-2 gene (1) -------------------------------------------------------------CT-TGTTTGAGCATATCTCTAGACTA-ATATATATATAT

BoMYB43-1 gene (1) -----------------------------GGAAATACAACAT--TGTCAGTATATTAGATA---TATTTGAGCACATCTCTAGACTATATAAAGTAAGAA

BoMYB43-2 gene (1) ---------------------------ATATACATTATAACT--TGTAAGTATATTAGGTACT-TGTTTGAGCATATCTCTAGACTA-ATAGATATATAT

Consensus (1) GGAAATACAACAT TGT AGTATATTAG TA TATTTGAGCATATCTCTAGACTA ATA AGATATAT

101 200

AtMYB43 gene (101) ATATATTTA------GCGAGAGAG-------------GGAGAGA-GATGGGGAGGCAACCATGTTGTGACAAAGTAGGGTTAAAGAAAGGGCCATGGACT

BnMYB43-1 gene (69) AAATATATAT------AGAGAGA--------------GGAGAGA-GATGGGGAGGCAACCATGTTGTGACAAAGTAGGCTTGAAGAAAGGCCCATGGACT

BnMYB43-2 gene (69) AAATATATA------GAGAGAGAG------------AGGAGAGA-GATGGGGAGGCAACCATGTTGTGACAAAGTAGGCTTGAAGAAAGGGCCATGGACT

BnMYB43-3 gene (38) ATATATATATAGA--GAGAGAGAGACAGAGGGTGCGAGGAGTGAAGATGGGGAGGCAGCCATGTTGTGACAAAGTAGGGCTGAAGAAAGGGCCATGGACA

BnMYB43-4 gene (70) ATATATAGA------GAGAGAGAGCCAGAGGGTGCGAGGAGTGAAGATGGGGAGGCAGCCATGTTGTGACAAAGTAGGGCTGAAGAAAGGGCCATGGACA

BrMYB43-1 gene (69) AAATATATAT------AGAGAGA--------------GGAGAGA-GATGGGGAGGCAACCATGTTGTGACAAAGTAGGCTTGAAGAAAGGCCCATGGACT

BrMYB43-2 gene (38) ATATATATATATATAGAGAGAGAGACAGAGGGTGCGAGGAGTGAAGATGGGGAGGCAGCCATGTTGTGACAAAGTAGGGCTGAAGAAAGGGCCATGGACA

BoMYB43-1 gene (67) AAATATATAT------AGAGAGA--------------GGAGAGA-GATGGGGAGGCAGCCATGTTGTGACAAAGTAGGCTTGAAGAAAGGGCCATGGACT

BoMYB43-2 gene (70) ATAGAGA--------GAGAGAGAGCCAGAGGGTGCGAGGAGTGAAGATGGGGAGGCAGCCATGTTGTGACAAAGTAGGGCTGAAGAAAGGGCCATGGACA

Consensus (101) ATATATATAT GAGAGAGAG AGGAGAGA GATGGGGAGGCAGCCATGTTGTGACAAAGTAGGGTTGAAGAAAGGGCCATGGACT

201 300

AtMYB43 gene (181) ATTGAAGAAGACAAGAAGCTCATCAACTTCATCCTCACCAATGGCCATTGCTGTTGGAGAGCTCTTCCCAAGCTTTCTGGTTTATTATCTC----TCTTT

BnMYB43-1 gene (148) GTTGAAGAAGACAAGAAGCTTATCAACTTCATCCTCACCAATGGCCAATGCTGTTGGAGAGCTCTTCCCAAGCTTTCTGGTCGATTTTCTC----TCCTT

BnMYB43-2 gene (150) GTTGAAGAAGACAAGAAGCTCATCAACTTCATCCTCACCAATGGCCAATGCTGTTGGAGAGCTCTTCCCAAGCTTTCTGGTCGATTTTCTC----TCTTT

BnMYB43-3 gene (136) GCTGAGGAAGACAAGAAGCTCATCAACTTCATCCTCACCAATGGCCACTGCTGTTGGAGAGCTCTTCCCAAGCTTTCTGGTATATTTTCT----TTCTTT

BnMYB43-4 gene (164) GCTGAGGAAGACAAGAAGCTCATCAACTTCATCCTCACCAATGGCCACTGCTGTTGGAGAGCTCTTCCCAAGCTTTCTGGTATATCTTCTCTCTTTCTTT

BrMYB43-1 gene (148) GTTGAAGAAGACAAGAAGCTTATCAACTTCATCCTCACCAATGGCCAATGCTGTTGGAGAGCTCTTCCCAAGCTTTCTGGTCGATTTTCTC----TCTTT

BrMYB43-2 gene (138) GCTGAGGAAGACAAGAAGCTCATCAACTTCATCCTCACCAATGGCCACTGCTGTTGGAGAGCTCTTCCCAAGCTTTCTGGTATATTTTCT----TTCTTT

BoMYB43-1 gene (146) GTTGAAGAAGACAAGAAGCTCATCAACTTCATCCTCACCAATGGCCAATGCTGTTGGAGAGCTCTTCCCAAACTTTCTGGTCGATTTTCTC----TCTTT

BoMYB43-2 gene (162) GCTGAGGAAGACAAGAAGCTCATCAACTTCATCCTCACCAATGGCCACTGCTGTTGGAGAGCTCTTCCCAAGCTTTCTGGTATATCTTCTCTCTTTCTTT

Consensus (201) GTTGAAGAAGACAAGAAGCTCATCAACTTCATCCTCACCAATGGCCA TGCTGTTGGAGAGCTCTTCCCAAGCTTTCTGGT TATTTTCTC TCTTT

301 400

AtMYB43 gene (277) CTCTTAACTTAACAAGACACCGTTTGTGATGGCTCATGTTTAGAAATTT-CAGTTTTGCTT-----------------------------CACACTTTTT

BnMYB43-1 gene (244) ATTTCCTCCTTAAACGACATCTTTTGGGATTTCTCATGTGTAAAAGTTCACTTTCTTGCTTCTTGTGTGTTTAGCCTTTCTCTACGCCAACTGACGCTTT

BnMYB43-2 gene (246) ATTTCCTCCTTAAACGACATCTTTTGGGATTTCTCATGTGTAAAAGTTCACTTTCTTGCTTCTTGTGTGTTTAGCCTTTCTCTACACCAACTGATGCTAT

BnMYB43-3 gene (232) CTTTTCTCCACATGCGACGTCCTT-GGGATTTCTCATGTGTAAAAAGTTCGTTTTTTTCTT---------------------------AACACATG-TGT

BnMYB43-4 gene (264) CTTTTCTCCATATGCGACGTCCTT-GGGATTTCTCATGTGTAAAAAGTT--TTTTTTTCTT---------------------------AACACATG-TGT

BrMYB43-1 gene (244) ATTTCCTCCTTAAACGACATCTTTTGGGATTTCTCATGTGTAAAAGTTCACTTTCTTGCTTCTTGTGTGTTTAGCCTTTCTCTACGCCAACTGACGCTTT

BrMYB43-2 gene (234) CTTTTCTCCACATGCGACGTCCTT-GGGATTTCTCATGTGTAAAAAGTTCGTTTTTTTCTT---------------------------AACACATG-TGT

BoMYB43-1 gene (242) ATTTCCTCCTTAAACGACATCTTTTGGGATTTCTCATGTGTAAAAGTTCACTTTCTTGCTTCTTGTGTGTTTAGCCTTTCTCTACACCAACTGATGCTAT

BoMYB43-2 gene (262) CTTTTCTCCATATGCGACGTCCTT-GGGATTTCTCATGTGTAAAAAGTT--TTTTTTTCTT---------------------------AACACACG-TGT

Consensus (301) CTTTTCTCCTTA ACGACATC TTTGGGATTTCTCATGTGTAAAAATTT CTTTTTTGCTT AACACATG T T

401 500

AtMYB43 gene (347) CTT-----TACCTTAAATCTTACC-GACGAAAACGATACTACTTTC----T-----TTGTTTTTTTGAAAAAAGTACATTTGTTGTTTTCATTCAAGTTT

BnMYB43-1 gene (344) TTT---GGTGCTATTTCTCTACACCGACGAAACTGATGCTATTTT-GATTTTTAGATACTCGTCTTTTAAAAGTT-CACTTCCTTAAAACGTCCAATTTT

BnMYB43-2 gene (346) TTTTTGGGTGCTATTTCTCTACACCGACGAAACTGATGCTCTTAT-----T-TAGGATCTCGTATTTTAAAAGTT-CACTTCCTTAAAACGTCCAATTTT

BnMYB43-3 gene (303) TTT-----TACATTTTCTCCTACCAAAAAAACGATATGCTATTTTTGATATATGGATTATCGTATCTTAAAAGTG-AACTTTCTTAAAACGTCCCCTTTT

BnMYB43-4 gene (333) TTT-----TACATTTTCTCTTACCAAAAAAATGATATGCTATTTTTGATATATGGATTATCGTATTTTAAAAGTT-AACTTTCTTAAAACGTCCCCTTTT

BrMYB43-1 gene (344) TTT---GGTGCTATTTCTCTACACCGACGAAACTGATGCTATTTT-GATTTTTAGATACTCGTCTTTTAAAAGTT-CACTTCCTTAAAACGTCCAATTTT

BrMYB43-2 gene (305) TTT-----TACATTTTCTCCTACCAAAAAAACGATATGCTATTTTTGATATATGGATTATCGTATCTTAAAAGTG-AACTTTCTTAAAACGTCCCCTTTT

BoMYB43-1 gene (342) TTTTTGGGTGCTATTTCTCTACACCGACGAAACTGATGCTCTTAT-----T-TAGGATCTCGTATTTTAAAAGTTTCACTTCCTTAAAACGTCCAATTTT

BoMYB43-2 gene (331) TTT-----TACATTTTCTCTTACCAAAAAAACGATATGCTATTTTTGATATATGGATTATCGTATTTTAAAAGTT-AACTTTCTTAAAACGTCCCCTTTT

Consensus (401) TTT TAC TTTTCTCTTACC GACGAAA GATGCTATTTT GAT T T GATT TCGTATTTTAAAAGTT CACTT CTTAAAACGTCCAATTTT

501 600

AtMYB43 gene (432) CCGATTTTTCAGTTATAGACCCATCTCACTCCTACTGATTCCTA----TTTTATTTACCTTTTTTTTATTCATTACCTA-GTAGTTTTCATGAGTTCTTT

BnMYB43-1 gene (439) ATTACTAGTAATTTCTATTTC--TCTACTTTCTTCTTGTCAGCAGTTTTTATAGTAATATTTTCTCTACTTTTGTGTAAATTAATTTGCATGAGTTATTT

BnMYB43-2 gene (439) ATTACTAGTAATTTATATTTC--TCTACTTTCTTCTTGTCAGCAGTTTTTGTAGTAATATTTTCTCTACTTTTGTGTAAATTAATTTGCATGAGTTATTT

BnMYB43-3 gene (397) ATTCCTACTGATTTCTATTTTA-TCTATTTTCTTGTTTTTGCTGACACTAATAATAATACTT-CTCTACCCTTTTTTAA-TTAATTTGCATGAGTTCTTT

BnMYB43-4 gene (427) ATTCCTACTGATTTCTATTTTA-TCTATTTTCTTGTTTTTGCTGACACTGATAATAATACTT-CTCTACC-TTTTTTAA-TTAATCTGCATGAGTTCTTT

BrMYB43-1 gene (439) ATTACTAGTAATTTCTATTTC--TCTACTTTCTTCTTGTCAGCAGTTTTTATAGTAATATTTTCTTTACTTTTGTGTAAATTAATTTGCATGAGTTATTT

BrMYB43-2 gene (399) ATTCCTACTGATTTCTATTTTA-TCTATTTTCTTGTTTTTGCTGACACTAATAATAATACTT-CTCTACCCTTTTTTAA-TTAATTTGCATGAGTTCTTT

BoMYB43-1 gene (436) ATTACTAGTAATTTATATTTC--TCTACTTTCTTCTTGTCAGCAGTTTTTATAGTAATATTTTCTCTACTTTTGTGTAAATTAATTTGCATTAGTTATTT

BoMYB43-2 gene (425) ATTCCTACTGATTTCTATTTTA-TCTATTTTCTTGTTTTTGCTGACACTGATAATAATACTT-CTCTACC-TTTTTTAA-TTAATCTGCATGAGTTCTTT

Consensus (501) ATTACTA T ATTTCTATTTC TCTA TTTCTTCTT TT CTA TTATA TAATATTTTCTCTACT TTTT TAA TTAATTTGCATGAGTTCTTT

601 700

AtMYB43 gene (527) AGACACAT--ATACTAATAG-GTTATGCGCCAATAATTAAGAAAA--TATAATT-TGATTTT--AAATATATTTTCAACGCAAGAAAATCACAATAATTC

BnMYB43-1 gene (537) TGGTATATGCATGCTAGTATAGATGTTCACCAACAATCAAATATACCTTTAGAAATTAGTTTCAAGAAACAATTTTGATGCAGGAGAATCATATGAATTC

BnMYB43-2 gene (537) TGGTATATGCATGCTAGTATAGATGTTCACCAACAATCAAATATACCTTTATAAATTATTTTCAAGAAACAATTTTGATGCAGAAGAATCATATGAATTC

BnMYB43-3 gene (494) TGCTATATCTATACTAGTATAGTTTTGCACCAA---TCAAATGAA--TTTAC----TATTTTC-AGTAATAGTTTCAATGCAGGAGAATAATATTCATTG

BnMYB43-4 gene (523) TGCTATATCTATACTAGTATAGTTTTGCACCAA--ATCAAATGAA--TTTAC----TATTTTTCAGTAATAGGTTCAATGCAGGAGAATCATATTCAATG

BrMYB43-1 gene (537) TGGTATATGCATGCTAGTATAGATGTTCACCAACAATCAAATATACCTTTAGAAATTAGTTTCAAGAAACAATTTTGATGCAGGAGAATCATATGAATTC

BrMYB43-2 gene (496) TGCTATATCTATACTAGTATAGTTTTGCACCAA---TCAAATGAA--TTTAC----TATTTTC-AGTAATAGTTTCAATGCAGGAGAATAATATTCATTG

BoMYB43-1 gene (534) TGGTATATGCATGCTAGTATAGATGTTCACCAACAATCAAATATACCTTTAGAAATTATTTTCAAGAAACAATTTTGATGCAGAAGAATCATATGAATTC

BoMYB43-2 gene (521) TGCTATATCTATACTAGTATAGTTTTGCACCAA---TCAAATGAA--TTTAC----TATTTTC-AGTAATAGTTTCAATGCAGGAGAATCATATTCATTG

Consensus (601) TG TATAT ATACTAGTATAGTT TGCACCAA AATCAAATAAA TTTA TTATTTTC AGAAATA TTTCAATGCAGGAGAATCATATTAATTC

701 800

AtMYB43 gene (619) TCACCTT-ACTTTTTGCAATGGAAT----ATATATCCCTTTTTCTGATTTCAAAAC-ATATTATTAG---------TT----TGTTAA--------TCAT

BnMYB43-1 gene (637) TTAACTTTCCTTTTTGAAATACAATATATATTTTAATATCGGTTGGACCTAAAAACAAAAGTCTCAAAACATATAATTAGACTATTAATCATAGGCTCAT

BnMYB43-2 gene (637) TTAACTTTCCTTTTTGAAATAGAAT----ATTTCTCTATCGGTTGGACCTAAAAACAAAAGTCTCAA---------------------------------

BnMYB43-3 gene (584) TTACCGT-CCTTTTTGAAATGGAAT----AATTCTCTATCTTTTCGACTTACGAA--ATCCGATCAA-----------------------------TCAT

BnMYB43-4 gene (615) ATACCGC-CCCTTTTGAAATGGAAG----AATTCTATATCTTTTCTACTGT-AAA--AACCTATCGA-----------------------------CCAT

BrMYB43-1 gene (637) TTAACTTTCCTTTTTGAAATACAATATATATTTTAATATCGGTTGGACCTAAAAACAAAAGTCTCAAAACATATAATTAGACTATTAATCATAGGCTCAT

BrMYB43-2 gene (586) TTACCGT-CCTTTTTGAAATGGAAT----AATTCTCTATCTTTTCGACTTACGAA--ATCCGATCAA-----------------------------TCAT

BoMYB43-1 gene (634) TTAACTTTCCTTTTTAAAATAGAAT----ATTTCTCTATCGGTTGGACCTAAAAACAAAAGTCTCAA---------------------------------

BoMYB43-2 gene (611) TTACCGT-CCTTTTTGAAATGGAAT----AATTCTCTATCTTTTCGACTTACAAA--ATCCGATCAA-----------------------------TCAT

Consensus (701) TTACCTT CCTTTTTGAAATGGAAT ATTTCTCTATCTTTT GACTTAAAAAC AAA TATCAA TCAT

801 900

AtMYB43 gene (692) AGTGTTTGGCTG----ATACAATCAGTGTTTGATTTTCGTGGTCCTCACACGCTATTATTTGGTTTT--TAATCAAT-----TTTAGTTTT---------

BnMYB43-1 gene (737) AGCGTCTGGCCCGAAAAGAAATATAGTGCTTGATATTCGTGGTCCTCACACGTTATTAGTTGATCTTGATAAAAAAAAATTGTTTTGTTTTTGGCTTTTT

BnMYB43-2 gene (700) ---------------A-AAAACATAGTGCTTGATATTCGTGGTCCTCACACGTTATTATTTGATCTTGATCAATTTTT-TTGTTTTGTTTTTGGCTTTTT

BnMYB43-3 gene (648) GGTGTTTGGCCCG-GACAAAAATCAATGTTTGATACTGGTGGTCCT-ACACGTTATTATTTGTTCTTGATAATTTTT---TGTTCTGTTTT--GTTTT--

BnMYB43-4 gene (678) GATGATTGGCCTTCGACTAAAG--AGTGGCCTCTACTGCCGCCCAT-TTATAT-AGTTTATGTTCATGAAAAATTATG--TGTTCTGAGAC--GTCTGGG

BrMYB43-1 gene (737) AGCGTCTGGCCCGAAAAGAAATATAGTGCTTGATATTCGTGGTCCTCACACGTTATTAGTTGATCTTGATAAAAAAAAATTGTTTTGTTTTTGGCTTTTT

BrMYB43-2 gene (650) GGTGTTTGGCCCG-GACAAAAATCAATGTTTGATACTGGTGGTCCT-ACACGTTATTATTTGTTCTTGATAATTTTT---TGTTCTGTTTT--GTTTT--

BoMYB43-1 gene (697) ---------------A-AAAACATAGTGCTTGATATTCGTGGTCCTCACACGTTATTATTTGATCTTGATCAATTTTT-TTGTTTTGTTTTTGGCTTTTT

BoMYB43-2 gene (675) GGTGTTTGGCCCG-GACAAAAATCAGTGTTTGATACTGGTGGTCCT-ACACGTTATTATTTGTTCTTGATAATTTTT---TGTTCTGTTTT--GTTTTGT

Consensus (801) GTGTTTGGCCCG A AAAA AGTG TTGATATTCGTGGTCCTCACACGTTATTATTTG TCTTGATAAATTTT TGTTTTGTTTT G TTT T

901 1000

AtMYB43 gene (772) ----------------------------------------ATAGTTTT-TAAGTTTAAATTAAATTGATCGTTGATTTAATTACTTTGTTTACA-AATTA

BnMYB43-1 gene (837) GTTTAGTGCTTCCTGCC-----AAAATATATT----TCGTACGGTTT--TAACTTTCAATCAATCTGATTGTTAATTTAATTACTTTGGTTAATTAACTA

BnMYB43-2 gene (783) GTTTAGTGCTTCCTGCCTAGTCAAAATATATT----TCGTATGGTTT--TAACTTTCAATCAGTCCAATTGTTAATTTAATTACTTTGGTTAATTAACTA

BnMYB43-3 gene (739) ---CACTTGTTCCTTCTTAATCAGTTTATCTC----TCGTATATTTAACTAGTTTCCAATCATTCTGATCGTTAATCTAGTTACTTTGGTTAATCAATTA

BnMYB43-4 gene (770) T--CACTTTTCAGGGAAAAGTCCGCGTAACCCCTATTTGTTCATTTTTCTC-TTTCCAATCATTCTGATCGTTAATCTAATTACTTTGGTTAATCAATTA

BrMYB43-1 gene (837) GTTTAGTGCTTCCTGCC-----AAAATATATT----TCGTATGGTTT--TAACTTTCAATCAATCTGATTGTTAATTTAATTACTTTGGTTAATTAACTA

BrMYB43-2 gene (741) ---CACTTGTTCCTTCTTAATCAGTTTATCTC----TCGTATATTTAACTAGTTTCCAATCATTCTGATCGTTAATCTAGTTACTTTGGTTAATCAATTA

BoMYB43-1 gene (780) GTTTAGTGCTTCCTTCCTAGTCAAAATATATT----TCGTATGGTTT--TAACTTTCAATCAGTCCAATTGTTAATGTAATTACTTTGGTTAATTAACTA

BoMYB43-2 gene (768) TTTCACTTGTTCCTTCTTATTCAGTTTATCTC----TCGTATATTTAACTAGTTTCCAATCATTCTGATCGTTAATCTAATTACTTTGGTTAATCAATTA

Consensus (901) TT A T TTCCT C TA TCA TAT T TCGTATAGTTT TAA TTTCAATCA TCTGATCGTTAAT TAATTACTTTGGTTAAT AATTA

1001 1100

AtMYB43 gene (830) AATGCAGGACTATTGCGGTGTGGTAAAAGCTGCAGATTAAGATGGATAAATTATTTAAGACCTGATTTAAAAAGAGGTCTTTTATCAGAATATGAAGAAC

BnMYB43-1 gene (926) AATGCAGGACTATTGAGGTGTGGTAAAAGCTGCAGATTAAGATGGATAAATTATTTGAGACCTGATTTAAAAAGAGGTCTTTTATCAGAAAATGAAGAGC

BnMYB43-2 gene (877) AATGCAGGACTATTGAGGTGTGGTAAAAGCTGCAGATTAAGATGGATAAATTATTTGAGACCTGATTTAAAAAGAGGTCTTTTATCTGAAAATGAAGAGC

BnMYB43-3 gene (832) AAT-CAGGACTCTTGAGGTGTGGAAAAAGCTGCAGACTAAGATGGATCAATTATTTGAGACCTGATTTAAAAAGAGGTCTTTTATCAGAATGTGAAGAAC

BnMYB43-4 gene (867) AAT-CAGGACTCTTGAGGTGTGGAAAAAGCTGCAGACTAAGATGGATCAATTATTTGAGACCTGATTTAAAAAGAGGTCTTTTATCAGAATGTGAAGAAC

BrMYB43-1 gene (926) AATGCAGGACTATTGAGGTGTGGTAAAAGCTGCAGATTAAGATGGATAAATTATTTGAGACCTGATTTAAAAAGAGGTCTTTTATCAGAAAATGAAGAGC

BrMYB43-2 gene (834) AAT-CAGGACTCCTGAGGTGCGGAAAAAGCTGCAGACTAAGATGGATCAATTATTTGAGACCTGATTTAAAAAGAGGTCTTTTATCAGATTGTGAAGAAC

BoMYB43-1 gene (874) AATGCAGGACTATTGAGGTGTGGTAAAAGCTGCAGATTAAGATGGATAAATTATTTGAGACCTGATTTAAAAAGAGGTCTTTTATCAGAAAATGAAGAGC

BoMYB43-2 gene (864) AAT-CAGGACTCTTGAGGTGTGGAAAAAGCTGCAGACTAAGATGGATCAATTATTTGAGACCTGATTTAAAAAGAGGTCTTTTATCAGAATGTGAAGAAC

Consensus (1001) AATGCAGGACTATTGAGGTGTGGTAAAAGCTGCAGATTAAGATGGATAAATTATTTGAGACCTGATTTAAAAAGAGGTCTTTTATCAGAATATGAAGAAC

1101 1200

AtMYB43 gene (930) AAAAGGTCATTAATCTCCATGCGCAACTTGGCAATAGGTTCATTCTCTTTCTCCTGAAAACAATTATTCT---------------------------ACA

BnMYB43-1 gene (1026) AGATGGTCATTGATCTTCATGCCCAACTTGGCAATAGGTTTATCCTCTTTTTATTCAAAATAATTGTTATTTCATATT-------------------ATA

BnMYB43-2 gene (977) AGATGGTCATTGATCTTCATGCCCAACTTGGCAATAGGTTTGCTCTCTTTTTCTTCAAAATAATTGTTATTTCATATT-------------------ATA

BnMYB43-3 gene (931) AGATGGTCATTGATCTTCATGCCCAACTTGGCAATAGGTTTATTCTCTTTCTCTTGAAAATCATTGTTATTCCATATTTTAAAAAATCCTCAAATTTATA

BnMYB43-4 gene (966) AGATGGTCATTGATCTTCATGCCCAACTTGGCAATAGGTTTATTCTCTTTCTCTGGAAAATCATTGTTATTCCATATTTTTAAAAATCCTCAAATTTATA

BrMYB43-1 gene (1026) AGATGGTCATTGATCTTCATGCCCAACTTGGCAATAGGTTTATCCTCTTTTTATTCAAAATAATTGTTATTTCATATT-------------------ATA

BrMYB43-2 gene (933) AGATGGTCATTGATCTTCATGCCCAACTTGGCAATAGGTTTATTCTCTTTCTCTTGAAAATCATTGTTATTCCATATTTTAAAAAATCCTCAAATTTATA

BoMYB43-1 gene (974) AGATGATCATTGATCTTCATGCCCAACTTGGCAATAGGTTTGCTCTCTTTTTCTTCAAAATAATTGTTATTTCATATT-------------------ATA

BoMYB43-2 gene (963) AGATGGTCATTGATCTTCATGCCCAACTTGGCAATAGGTTTATTCTCTTTCTCTGGAAAATCATTGTTATTCCATATTTTTAAAAATCCTCAAATTTATA

Consensus (1101) AGATGGTCATTGATCTTCATGCCCAACTTGGCAATAGGTTTATTCTCTTTCTCTTGAAAATAATTGTTATT CATATT ATA

1201 1300

AtMYB43 gene (1003) AACAATAATTT-ACTTACTTTAAC-----------------------------TATGACGTTCTAACTAACTCATAATA----AAAGTATATAAAGTTTA

BnMYB43-1 gene (1107) AACA-TAATTT-ACTTACCTTAT-------------------------------TTGACATTGTAACTAACTCATAATATAATGAAATGTTTAAA---GA

BnMYB43-2 gene (1058) AACA-TAATTT-ACTTACGTTAAC------------------------------TTGACATTGTAACTAACTAATAATATAATAAAATATTTAAA---GA

BnMYB43-3 gene (1031) AACAGTAATTTTACCTACCTTTTAAAAAAAAAACTAATTTTACCTGCCTTAACTTTGATGTTGTAACTAACTCGTAATA----AAAATATAAAAA---TA

BnMYB43-4 gene (1066) AACAGTAATTTTACCTACCTTTTT-AAAAAAAACTAATTTCACCTGCCA-AACTTTTATGTTGTAACTAACTCGTAATA----AAAATATAAAAAA--TA

BrMYB43-1 gene (1107) AACA-TAATTT-ACTTACCTTAT-------------------------------TTGACATTGTAACTAACTCATAATATAATGAAATGTTTAAA---GA

BrMYB43-2 gene (1033) AACAGTAATTTTACCTACCTTTTTAAAAAAAAACTAATTTTACCTGCCTTAACTTTGATGTTGTAACTAACTCGTAATA----AAAATATAAAAA---TA

BoMYB43-1 gene (1055) AACA-TAATTT-ACTTACGTTAAC------------------------------TTGACATTGTAACTAACTAATAATATAATAAAATATTTAAA---GA

BoMYB43-2 gene (1063) AACAGTAATTTTACCTACCTTTT--AAAAAAAACTAATTTTACCTGCCA-AACTTTGATGTTGTAACTAACTCGTAATA----AAAATATAAAAAA--TA

Consensus (1201) AACA TAATTT ACTTACCTTAT TTTGACGTTGTAACTAACTCATAATA AAAATATATAAA TA

1301 1400

AtMYB43 gene (1069) TATATATGACCATTATATTACAATATTCTC-AAGTTATCGGAGAAACACAAAAGTCGTATTTAGTCGTATTTAGACTCGAACTTTATCTCATCTTT-CGC

BnMYB43-1 gene (1171) T---TATGGACATTAGATTTCAATATTA---AAGGTATTGGAGAAGCACAAAAGTCATTATATGACGCA----------AACTTTATCACTTTTTT-TGC

BnMYB43-2 gene (1123) T---TATGGCCATTTGATTTCAATATTATTAAGGGTATTGGAGAAACACAAAAGTCATTATATGACGCAAATT------AACTTTATCACTTTTTT--GC

BnMYB43-3 gene (1124) T---TGTGGCCATTAGATTTCAATATTATT-AAGGTCACGGAGGAACACAAAAGTCACAATAGAACACTGAAC-ACGCAAACTTTACTATTTTTTTCTGT

BnMYB43-4 gene (1158) T---TGTGGCCATTAGACTTCAATATTATT-AAGGTCTCGGAGGAACACAAAAGTCACAATAGAACACTGAAC-ACGCAAACTTTACTATTTCTT-CTGT

BrMYB43-1 gene (1171) T---TATGGACATTAGATTTCAATATTA---AAGGTATTGGAGAAGCACAAAAGTCATTATATGACGCA----------AACTTTATCACTTTTTT-TGC

BrMYB43-2 gene (1126) T---TGTGGCCATTAGATTTCAATATTATT-AAGGTCACGGAGGAACACAAAAGTCACAATAGAACACTGAAC-ACGCAAACTTTACTATTTTTTTCTGT

BoMYB43-1 gene (1120) T---TATGGCCATTTGATTTCAATATTATTAAGGGTATTGGAGAAACACAAAAGTCATTATATGACGCAAATT------AACTTTATCACTTTTTT--GC

BoMYB43-2 gene (1154) T---TGTGGCCATTAGACTTCAATATTATT-AAGGTCTCGGAGGAACACAAAAGTCACAATAGAACACTGAAC-ACGCAAACTTTACTATTTCTT-CTGT

Consensus (1301) T TATGGCCATTAGATTTCAATATTATT AAGGTATCGGAGAAACACAAAAGTCATAATA GACGCA A AC C AACTTTATCACTTTTTT TGC

1401 1500

AtMYB43 gene (1167) ACGAAAAAGATTACCTAT--CACATCTCTTATTTAG---TCATATT---TTTTAAGCCGAGTA-------GTTTAATTTAGTTGGTCTGATGAAAATGAC

BnMYB43-1 gene (1254) ATGAAAGAGATTGGCTAATCCACATTTT-TAATTAC---TCATATTTTCTTTCAAACCCAATA-------GTTTAATTAAGTCGGTGTGATGAAAATGTT

BnMYB43-2 gene (1212) ATGAAAGAGATTGGCTAATCCACATCTT-TATTTAC---TCAAATTTTCTTTTTAACCCCAGT----A--GTTTAATTAAGTCGGTGTGATGAAAATGTT

BnMYB43-3 gene (1219) ATGAAAGAGATTGGCCAATCTACATCTCATATTTAAATTTAATCGTGCGTTTTTAACCACGTACGGTA--GTTTAATTTAGTAAGTCTGATGATAATGAC

BnMYB43-4 gene (1252) ATGAAAGAGATTGGCCAATCTACATCTCATATTTAAATTTAATCGTGCGTTTTTAACCACGTACGGTA--GTTTAATTTAGTAGGTCTGATGATAATGAC

BrMYB43-1 gene (1254) ATGAAAGAGATTGGCTAATCCACATTTT-TAATTAC---TCATATTTTCTTTCAAACCCAATAT---A--GTTTAATTAAGTCGGTGTGATGAAAATGTT

BrMYB43-2 gene (1221) ATGAAAGAGATTGGCCAATCTACATCTCATATTTAAATTTAATCGTGCGTTTTTAACCACGTACGGTA--GTTTAATTTAGTAAGTCTGATGATAATGAC

BoMYB43-1 gene (1209) ATGAAAGAGATTGGCTAATCCACATCTT-TATTTAC---TCATATTTTCTTTTTAACCCCAGT----A--CTTTAATTAAGTCGGTGTGATGAAAATGTT

BoMYB43-2 gene (1248) ATGAAAGAGATTGGCCAATCTACATCTCATATTTAAATTTAATCGTGCGTTTTTAACCACGTACGGTATAGTTTAATTTAGTAGGTCTGATGATAATGAC

Consensus (1401) ATGAAAGAGATTGGCTAATCCACATCTC TATTTA TCATATT TTTTTAACC CGTA A GTTTAATTTAGT GGTCTGATGAAAATGAC

1501 1600

AtMYB43 gene (1252) TGAACAGTCGAAATATTTACTAAATTTTAAGCTTATATGATTTT-GAATCTTTAACTTTTATTTTGTGTGTGTGTGCAGATGGTCAAAGATTGCCTCTCA

BnMYB43-1 gene (1343) TGAACAGTCGAAATCATTAGTGAAATTATCACTTATAAAATTTTCAAATATTTTATTTTTATTTTT-------GTGCAGATGGTCAAAGATTGCCTCTCA

BnMYB43-2 gene (1302) TGAACAGTCGAAATCATTAGTTAAATTCACACTTATAAAATTTTCAAATATTTTATTTTTATTTTTC------GTGCAGATGGTCAAAGATTGCCTCTCA

BnMYB43-3 gene (1317) TGAACAGTCGAAATCATGAGTTAA--TTAAACTTACTTAAGTATCGAATC----ATTTTAATTTTT-------GTGCAGATGGTCAAAGATTGCCTCTCA

BnMYB43-4 gene (1350) TGAACAGTCGAAATCATGAGTTAA--TTAAACTTA----AGTTTCGAATC----ATTTTAATTTTT-------GTGCAGATGGTCAAAGATTGCCTCTCA

BrMYB43-1 gene (1345) TGAACAGTCGAAATCATTAGTGAAATTATCACTTATAAAATTTTCAAATATTTTATTTTTATTTTT-------GTGCAGATGGTCAAAGATTGCCTCTCA

BrMYB43-2 gene (1319) TGAACAGTCGAAATCATGAGTTAA--TTAAACTTACTTAAGTATCGAATC----ATTTTAATTTTT-------GTGCAGATGGTCAAAGATTGCCTCTCA

BoMYB43-1 gene (1299) TGAACAGTCGAAATCATTAGTTAAAATCACACTTATAAAATTTTCAAATATTTTATTTTTATTTTTT------GTGCAGATGGTCAAAGATTGCCTCTCA

BoMYB43-2 gene (1348) TGAACAGTCGAAATCATGAGTTAA--TTAAACTTACTTAAGTTTCGAATC----ATTTTAATTTTT-------GTGCAGATGGTCAAAGATTGCCTCTCA

Consensus (1501) TGAACAGTCGAAATCATTAGTTAA TTAAACTTATA AATTTTCGAATCTTT ATTTTTATTTTT GTGCAGATGGTCAAAGATTGCCTCTCA

1601 1700

AtMYB43 gene (1351) TCTACCAGGAAGAACTGATAATGAAATAAAGAACCATTGGAACACACACATAAAGAAAAAGCTAAGGAAAATGGGTATTGACCCTTTGACCCATAAGCCT

BnMYB43-1 gene (1436) TCTACCAGGAAGAACTGATAATGAAATTAAGAACCATTGGAACACACACATAAAGAAAAAACTAAGGAAGATGGGTATTGACCCTATGACCCATAAGCCT

BnMYB43-2 gene (1396) TCTACCAGGAAGAACTGATAATGAAATTAAGAACCATTGGAACACACACATAAAGAAAAAACTAAGGAAGATGGGTATTGACCCTATGACCCATAAGCCT

BnMYB43-3 gene (1404) TCTACCAGGAAGAACTGATAATGAAATAAAGAACCATTGGAACACACACATAAAGAAAAAACTAAGGAAGATGGGCATTGACCCTATGACCCATAAGCCT

BnMYB43-4 gene (1433) TCTACCAGGAAGAACTGATAATGAAATAAAGAACCATTGGAACACACACATAAAGAAAAAACTAAGGAAGATGGGCATTGACCCTATGACCCATAAGCCT

BrMYB43-1 gene (1438) TCTACCAGGAAGAACTGATAATGAAATTAAGAACCATTGGAACACACACATAAAGAAAAAACTAAGGAAGATGGGTATTGACCCTATGACCCATAAGCCT

BrMYB43-2 gene (1406) TCTACCAGGAAGAACTGATAATGAAATAAAGAACCATTGGAACACACACATAAAGAAAAAACTAAGGAAGATGGGCATTGACCCTATGACCCATAAGCCT

BoMYB43-1 gene (1393) TCTACCAGGAAGAACTGATAATGAAATTAAGAACCACTGGAACACACACATAAAGAAAAAACTAAGGAAGATGGGTATTGACCCTATGACCCATAAGCCT

BoMYB43-2 gene (1435) TCTACCAGGAAGAACTGATAATGAAATAAAGAACCATTGGAACACACACATAAAGAAAAAACTAAGGAAGATGGGCATTGACCCTATGACCCATAAGCCT

Consensus (1601) TCTACCAGGAAGAACTGATAATGAAATAAAGAACCATTGGAACACACACATAAAGAAAAAACTAAGGAAGATGGGTATTGACCCTATGACCCATAAGCCT

1701 1800

AtMYB43 gene (1451) CTCTCTGAACAAGAAGCTTC---ACAACAAGCTCAAGGGAGAAAGAAAAGTTTAGTGCCTCATGATGACAAGAACCCAAAACAAG------A---TCAAC

BnMYB43-1 gene (1536) CTCTCTGAACAAGACGGTGCGCAACAACAAGCTCAGGGGAGAAAGAAAAGCTTAGTGTCTCGTGATGAGAAGAACACAAACCAAGGCCAAGAAGATCAAC

BnMYB43-2 gene (1496) CTCTCTGAACAAGACGGTGCGCAACAACAAGCTCAGGGGATAAAGAAAAGCTTAGTGTCTCGTGATGAGAAGAACACAAACCAAGGCCAAGAAGATCAAC

BnMYB43-3 gene (1504) CTCTCTGAACAAGACGGGTC---ACAGCAAGCTCAAGGGAGCAAGA---GTTTAGTGCCTCATGATGACAAGAACAAAAAACAAGACCAAGAAGATAAAC

BnMYB43-4 gene (1533) CTCCCTGAACAAGACGGTTC---ACAGCAAGCTCAAGGGAGCAAGA---GTTTAGTGCCTCATGATGACAAGAACAAAAAACAAGACCAAGAAGATAAAC

BrMYB43-1 gene (1538) CTCTCTGAACAAGACGGTGCGCAACAACAAGCTCAGGGGAGAAAGAAAAGCTTAGTGTCTCGTGATGAGAAGAACACAAACCAAGGCCAAGAAGATCAAC

BrMYB43-2 gene (1506) CTCCCTGAACAAGACGGGTC---ACAGCAAGCTCAAGGGAGCAAGA---GTTTAGTGCCTCATGATGACAAGAACAAAAAACAAGACCAAGAAGATAAAC

BoMYB43-1 gene (1493) CTCTCTGAACAAGAAGGTGCGCAGCAACAAGCTCAGGGGACAAAGAAAAGCTTAGTGTCTCGTGATGAGAAGAAAAGAAACCAAGGCCAAGAAGATCAAC

BoMYB43-2 gene (1535) CTCCCTGAACAAGACGGTTC---ACAGCAAGCTCAAGGGAGCAAGG---GTTTAGTGCCTCATGATGACAAGAACAAAAAACAAGACCAAGAAGATAAAC

Consensus (1701) CTCTCTGAACAAGACGGTTC ACAACAAGCTCAAGGGAGAAAGAAAAGTTTAGTGCCTCATGATGACAAGAACA AAAACAAG CCAAGAAGATCAAC

1801 1900

AtMYB43 gene (1539) AA---ACCAAAGACGAACAAGA---ACAACATCAATTAGAGCAAGCTTTGGAGAAGAACAACACATCAGTGT---CTGGTGATGGGTTTTGCATTGATGA

BnMYB43-1 gene (1636) AACAAACTAGAAAAGAAGAAGATCAACAACATCAACTAGGGAAAGATTTGGAGAAGAACAACACACCCATTTCTACTGATGATGGGTTTTGCATTGATGA

BnMYB43-2 gene (1596) AACAAACTAGAAAAGAAGAAGATCAACAACATCAACTAGGGAAAGATTTGGAGAAGAACAACACACCCATTTCTACTGATGATGGGTTTTGCATTGATGA

BnMYB43-3 gene (1598) AA---ACTAAAGAAAAACAAGA---ACACCATCAACTAGGGATAGATTTGGACAAGAGCAACAAAACAATATCTACTGATGATGGGTTTTGCATTGATGA

BnMYB43-4 gene (1627) AA---ACTAAAGAAAAACAAGA---ACACCATCAACTAGGGATAGATTTGGACAAGAGCAACAAAACAATATCTACTGATGATGGGTTTTGCATTGATGA

BrMYB43-1 gene (1638) AACAAACTAGAAAAGAAGAAGATCAACAACATCAACTAGGGAAAGATTTGGAGAAGAACAACACACCCATTTCTACTGATGATGGGTTTTGCATTGATGA

BrMYB43-2 gene (1600) AA---ACTAAAGAAAAACAAGA---ACACCATCAACTAGGGATAGATTTGGACAAGAGCAACAAAACAATATCTACTGATGATGGGTTTTGCATTGATGA

BoMYB43-1 gene (1593) AA---ACTAGAAAAGAAGAAGATCAACAACATCAACTAGGGAAACATTTGGACAAGAACAACACACCCATTTCTACTGATGATGGGTTTTGCATTGATGA

BoMYB43-2 gene (1629) AA---ACTAAAGAAAAACAAGA---ACACCATCAACTAGGGATAGATTTGGACAAGAGCAACAAAACAATATCTACTGATGATGGGTTTTGCATTGATGA

Consensus (1801) AA ACTAAAGAAGAACAAGA ACAACATCAACTAGGGAAAGATTTGGACAAGAACAACACA CAAT TCTACTGATGATGGGTTTTGCATTGATGA

1901 2000

AtMYB43 gene (1630) AGTCCCATTGCTCAATCCACATGAGATCTTGATCGACATCTCTTCTTCTCATCATCATCATTCTAATGATGATAATGTCAATATCAACACTAGTAAATTT

BnMYB43-1 gene (1736) TATCCCATTACTCAGTCCACATGAGATATTTGTGGACATCCCTTCTGCTCACCATCATA---CTAGTGATGATAATGCCAACATTAACATTAGTAACTCC

BnMYB43-2 gene (1696) TATCCCATTACTCAGTCCACATGAGATATTTGTGGACATCTCTTCTGCTCACCATCATA---CTAGTGATGATAATGCCAACATTAACATTAGTAACTCC

BnMYB43-3 gene (1692) AGTCCCGTTGCTCGATCCACATGAGATATTACTGGACATCTCTTTTGCTGATCATCATA---CTAGTGATGAAAATGCTCACATTAACATTAGTAACTCC

BnMYB43-4 gene (1721) AGTCCCGTTGCTCGATCCACATGAGATATTACTGGACATCTCTTTTGCTCAGCATCATA---CTAGTGATGAAAATGCTCACATTAACATTAGTAACTCC

BrMYB43-1 gene (1738) TATCCCATTACTCAGTCCACATGAGATATTTGTGGACATCTCTTCTGCTCACCATCATA---CTAGTGATGATAATGCCAACATTAACATTAGTAACTCC

BrMYB43-2 gene (1694) AGTCCCGTTGCTCGATCCACATGAGATATTACTGGACATCTCTTTTGCTGATCATCATA---CTAGTGATGAAAATGCTCACATTAACATTAGTAACTCC

BoMYB43-1 gene (1690) TATCCCATTACTCAATCCACATGAGATATTTGTGGACATCTCTTCTGCTCACCATCATA---CTAGTGATGGTAATGCCAACATTAACATTAGTAACTCC

BoMYB43-2 gene (1723) AGTCCCGTTGCTCGATCCACATGAGATATTACTGGACATCTCTTTTGCTCAGCATCATA---CTAGTGATGAAAATGCTCACATTAACATTAGTAACTCC

Consensus (1901) AGTCCCATTGCTCAATCCACATGAGATATT TGGACATCTCTTCTGCTCA CATCATA CTAGTGATGATAATGCCAACATTAACATTAGTAACTCC

2001 2100

AtMYB43 gene (1730) ACTTCTCCTTCTTCCTCTTCCTCTTCTACGTCGTCGTGTATATCATCAGTAGTACCGGGTGATGAGTTCTCCAAGTTTTTTGATGAAATGGAGATTCTTG

BnMYB43-1 gene (1833) ACTTCACCTTCTTCGTCTTCCTCTTCTACCTCGTCGTGCATATCATCACTA---CCGGGTGATGAGTTCTCAAAGTTTCTTGATGAAATGGAGATTGTTG

BnMYB43-2 gene (1793) ACTTCACCTTCTTCGTCTTCCTCTTCTACTTCGTCATGCATATCATCACTA---CCGGGTGATGAGTTCTCAAAGTTTCTTGATGAAATGGAGATTGTTG

BnMYB43-3 gene (1789) ACTTCTCCTTCTTC------CTCTTCTACCTCGTCGTGTATATCATCAGTA---CCGGGTGAGGAGTTCTCGAAGTTTCTTGATGAAATGGACACTATTG

BnMYB43-4 gene (1818) ACTTCTCCTTCTTC------CTCTTCTACCTCGTCGTGTATATCATCAGTA---CCGGGTGAGGAGTTCTCGAAGTTTCTTGATGAAATGGAGACTATTG

BrMYB43-1 gene (1835) ACTTCACCTTCTTCGTCTTCCTCTTCTATCTCGTCGTGCATATCATCACTA---CCGGGTGATGAGTTCTCAAAGTTTCTTGATGAAATGGAGATTGTTG

BrMYB43-2 gene (1791) ACTTCTCCTTCTTC------CTCTTCTACCTCGTCGTGTATATCATCAGTA---CCGGGTGAGGAGTTCTCGAAGTTTCTTGATGAAATGGACACTATTG

BoMYB43-1 gene (1787) ACTTCACCATCTTCGTCTTCCTCTCCTACCTCGTCGTGCATATCATCACTA---CCAGGTGATGAGTTCTCAAAGTTTCTTGATGAAATGGAGATTGTTG

BoMYB43-2 gene (1820) GCTTCTCCTTCTTC------CTCTTCTACCTCGTCGTGTATATCATCAGTA---CCGGGTGAGGAGTTCTCGAAGTTTCTTGATGAAATGGAGACTATTG

Consensus (2001) ACTTCTCCTTCTTC TCTTCCTCTTCTACCTCGTCGTGTATATCATCAGTA CCGGGTGATGAGTTCTC AAGTTTCTTGATGAAATGGAGATT TTG

2101 2200

AtMYB43 gene (1830) ACCTCAAGTGGCTTTCATCCGATGATTCTTTAGGGGACGATATTAG---CAAAGACGGCAAGTTCAACAACAGTACTGTTGATACGATGAACTTGTGGGA

BnMYB43-1 gene (1930) ACCTCAAGTGGCTCTCATCCGATGATTCATCAGGGGGTAACATTAATTACAACGACGGCAAGTTCAACAACAAT---GTTGATACGATGAGCTTGTGGGA

BnMYB43-2 gene (1890) ATCTCAAGTGGCTCTCATCCGATGATTCATCAGGGGGTGACATTAATTACAACGACGGCAAGTTCAACAACAAT---GTTGATACGATGAGCTTGTGGGA

BnMYB43-3 gene (1880) ACCTCAAGTGGCTCTCATCCGATCATTCATCAGAGGATAATATTATTTACAAAGACGACAAGTTCCACAACAAT---GTTGATACGATGAACTTGTGGGA

BnMYB43-4 gene (1909) ACCTCAAGTGGCTCTCATCCGATCATTCATCAGGGGATAATATTATTTACAAAGACGACAAGTTCCACAACAAT---GTTGATACGATGAACTTGTGGGA

BrMYB43-1 gene (1932) ACCTCAAGTGGCTCTCATCCGATGATTCATCAGGGGGTAACATTAATTACAACGACGGCAAGTTCAACAACAAT---GTTGATACGATGAGCTTGTGGGA

BrMYB43-2 gene (1882) ACCTCAAGTGGCTCTCATCCGATCATTCATCAGAGGATAATATTATTTACAAAGACGACAAGTTCCACAACAAT---GTTGATACGATGAACTTGTGGGA

BoMYB43-1 gene (1884) ACCTCAAGTGGCTCTCATCCGATGATTCGTCAGGGGGTGACATTAATTACAAAGACGGAAAGTTCAATAACAAT---GTTGATACGATGAACTTGTGGGA

BoMYB43-2 gene (1911) ACCTCAAGTGGCTCTCATCCGATCATTCATCAGGGGATAATATTATTTACAAAGACGACAAGTTCCACAACAAT---GTTGATACGATGAACTTGTGGGA

Consensus (2101) ACCTCAAGTGGCTCTCATCCGATGATTCATCAGGGGATAATATTA TTACAAAGACGGCAAGTTCAACAACAAT GTTGATACGATGAACTTGTGGGA

2201 2300

AtMYB43 gene (1927) CATCAATGATTTGAGCAGCTTGGATATGTTT-ATGAATGAACATGATGATGGTTTTATTGGAAATGGAAATGGATGTTCAAGAATGGTTTTAGATCAAGA

BnMYB43-1 gene (2027) CATCAATGATTTGAGCAGCTTGGAATTGTTT-ATGAATGATCATGATGATAGTGTGGTTG-------AAGCGGATGTTCAAGAATGGTTTTAGATCATGA

BnMYB43-2 gene (1987) CATCAATGATTTGAGCAGCTTGGAATTGTTTTATGAATGATCATGATGATAGTGTGGTTG-------AAGCGGATGTTCAAGAATGGTTTTAGATCATGA

BnMYB43-3 gene (1977) CATAAATTATTTGAGCAGCTTGGAATTGTTT-ATGAATGATCATGATGATGGTTTTGTTGG------AAGCGGATGTTCAAGAATGGTTT-AGATCAATA

BnMYB43-4 gene (2006) CATAAATTATTTGAGCAGCTTGGAATTGTTT-ATGAATGATCATGATGATGGTTTTGTTGG------AAGCGGATGTTCAAGAATGGTTT-AGATCAATA

BrMYB43-1 gene (2029) CATCAATGATTTGAGCAGCTTGGAATTGTTT-ATGAATGATCATGATGATAGTGTGGTTG-------AAGCGGATGTTCAAGAATGGTTTTAGATCATGA

BrMYB43-2 gene (1979) CATAAATTATTTGAGCAGCTTGGAATTGTTT-ATGAATGATCATGATGATGGTTTTGTTGG------AAGCGGATGTTCAAGAATGGTTT-AGATCAATA

BoMYB43-1 gene (1981) CATCAATGATTTGAGCAGCTTGGAATTGTTT-ATGAATGATCACGATGATAGTGTGGTTGG------AAGCGGATGTTCAAGAATGGTTTTAGATCATGA

BoMYB43-2 gene (2008) CATAAATTATTTGAGCAGCTTGGAATTGTTT-ATGAATGATCATGATGATGGTTTTGTTGG------AAGCGGATGTTCAAGAATGGTTT-AGATCAATA

Consensus (2201) CATCAATGATTTGAGCAGCTTGGAATTGTTT ATGAATGATCATGATGATGGTTTTGTTGG AAGCGGATGTTCAAGAATGGTTTTAGATCAAGA

2301 2400

AtMYB43 gene (2026) TTCATGGACATTTGATCTCCTCTAGCTTTTAGTTTTTTCTTGATGTTTTTTTAATTAGATTTTTTTTTTTTGTTCTTGAAGTAGTGGGGGGATTTGGGGT

BnMYB43-1 gene (2119) TTCATGGACATTTGATCTTCTTTAACTTTTAGTTTTTTTTT--------CTTCTCATG-TTGTTTTTAATTGTTTTT-GTTT----GTTTG-TTTGGGTT

BnMYB43-2 gene (2080) TTCATGGACATTTGATCTTCTTTAACTTTTAGTTTTTTTTT-------TCTTCTCTTG-TTGTTTTTAATTGTTTTT-GTTT----GTTTG-TTTGGGTT

BnMYB43-3 gene (2069) TACATGACCATTTTACTTTCAGTTTTTTTTT-CATGTTGTT--------TTTAATTAG-TTCTTTTTGTTTGTTTTCGAAGT----GGGCGATTTGGATT

BnMYB43-4 gene (2098) TTCATGACCATTTTACTTTCAGTTTTTTTTTTCATGTTGTT--------TTTAATTAG-TTCTTTTTGTTTGTTTTCGAAGT----GGGCGATTCGGATT

BrMYB43-1 gene (2121) TTCATGGACATTTGATCTTCTTTAACTTTTAGTTTTTTTTT--------CTTCTCATG-TTGTTTTTAATTGTTTTT-GTTT----GTTTG-TTTGGGTT

BrMYB43-2 gene (2071) TACATGACCATTTTACTTTCAGTTTTTTTTTTCATGTTGTT--------TTTAATTAG-TTCTTTTTGTTTGTTTTCGAAGT----GGGCGATTTGGATT

BoMYB43-1 gene (2074) TTCATGGACATTTGATCTTCTTTAACTTTTAGTTTTTTTTT--------CTTCTCTTG-TTGTTTTTAATTGTTTTTTGTTT----GTTTG-TTTGGGTT

BoMYB43-2 gene (2100) TTCATGACCATTTTACTTTCAGTTTTTTTTTTCATGTTGTT--------TTTAATTAG-TTCTTTTTGTTTGTTTTCGAAGT----GGGCGATTTGGATT

Consensus (2301) TTCATGGACATTTGATCTTCT TA CTTTTAGTTTTTT TT TTTAATTAG TT TTTTT TTTGTTTTTGAAGT GGG GATTTGGGTT

2401 2500

AtMYB43 gene (2126) TGGC-TTGGTGTTGCAGTTTAGTGGATTAT--GGTGTACAA-TACAT--GTAAT-AAAGATTGCAGTTGTGGACAGATCGTTTTTTCTGTTTTTTTTCCT

BnMYB43-1 gene (2204) TGGCCTTGGTG----------GTGGATGATTAGGTGTAAAAGTACATATGTAATTAAAGATTGCAATTGTGAACATAC--TTTTCTCTGTTTAACCTCGT

BnMYB43-2 gene (2166) TGGCCTTGGTGTTGCAGTTTAGTGGATGATTAGGTGTAAAAGTACATATGTAATTAAAGATTGCAATTGTGAACATAC--CTTTCACTG-----------

BnMYB43-3 gene (2155) TGGCCTTGGTGTTGCAATTTAGTGGATGATTAGGTGTAAAA-TACATATGTAAT-AAATATTGCAATTGTGAACACAC--TTTTCTC-------------

BnMYB43-4 gene (2185) TGGCCGTGGTGTTGCAATTTAGTGGATGATTAGGTGTAAAA-TACATATGTAAT-AAAGATTGCAATTGTGAAC--------------------------

BrMYB43-1 gene (2206) TGGCCTTGGTG----------GTGGATGATTAGGTGTAAAAGTACATATGTAATTAAAGATTGCAATTGTGAACATAC--TTTTCTCTGTTTAACCTCGT

BrMYB43-2 gene (2158) TGGCCTTGGTGTTGCAATTTAGTGGATGATTAGGTGTAAAA-TACATATGTAAT-AAATATTGCAATTGTGAACATAC--TTTTCTC-------------

BoMYB43-1 gene (2160) TGGCCTCGGTGTTGCAGTTTAGTGGATGATTAGGTGTAAAAGTACATATGTAATTAAAGATTACAATTGTGAACATAC--TTTTCTCTGTTTATTTTGAC

BoMYB43-2 gene (2187) TGGCCGTGGTGTTGCAATTTAGTGGATGATTAGGTGTAAAA-TACATATGTAAT-AAAGATTGCAATTGTGAAC--------------------------

Consensus (2401) TGGCCTTGGTGTTGCA TTTAGTGGATGATTAGGTGTAAAA TACATATGTAAT AAAGATTGCAATTGTGAACATAC TTTTCTCTG

2501 2551

AtMYB43 gene (2219) TCACTTACGAAATATTTCTTTGGAAAAATATATATAAAACACCATTAAAGT

BnMYB43-1 gene (2292) AT-------------------------------------------------

BnMYB43-2 gene (2253) ---------------------------------------------------

BnMYB43-3 gene (2238) ---------------------------------------------------

BnMYB43-4 gene (2257) ---------------------------------------------------

BrMYB43-1 gene (2294) AT-------------------------------------------------

BrMYB43-2 gene (2241) ---------------------------------------------------

BoMYB43-1 gene (2258) AT-------------------------------------------------

BoMYB43-2 gene (2259) ---------------------------------------------------

Consensus (2501)

**(b)**


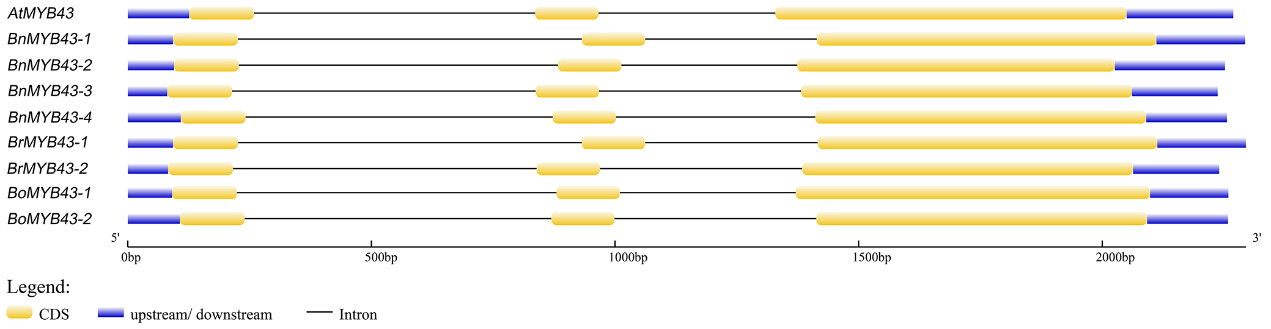


**(c)**

1 90

AtMYB43 (1) MGRQPCCDKVGLKKGPWTIEEDKKLINFILTNGHCCWRALPKLSGLLRCGKSCRLRWINYLRPDLKRGLLSEYEEQKVINLHAQLGNRWS

BnMYB43-1 (1) MGRQPCCDKVGLKKGPWTVEEDKKLINFILTNGQCCWRALPKLSGLLRCGKSCRLRWINYLRPDLKRGLLSENEEQMVIDLHAQLGNRWS

BrMYB43-1 (1) MGRQPCCDKVGLKKGPWTVEEDKKLINFILTNGQCCWRALPKLSGLLRCGKSCRLRWINYLRPDLKRGLLSENEEQMVIDLHAQLGNRWS

BnMYB43-2 (1) MGRQPCCDKVGLKKGPWTVEEDKKLINFILTNGQCCWRALPKLSGLLRCGKSCRLRWINYLRPDLKRGLLSENEEQMVIDLHAQLGNRWS

BoMYB43-1 (1) MGRQPCCDKVGLKKGPWTVEEDKKLINFILTNGQCCWRALPKLSGLLRCGKSCRLRWINYLRPDLKRGLLSENEEQMIIDLHAQLGNRWS

BnMYB43-3 (1) MGRQPCCDKVGLKKGPWTAEEDKKLINFILTNGHCCWRALPKLSGLLRCGKSCRLRWINYLRPDLKRGLLSECEEQMVIDLHAQLGNRWS

BrMYB43-2 (1) MGRQPCCDKVGLKKGPWTAEEDKKLINFILTNGHCCWRALPKLSGLLRCGKSCRLRWINYLRPDLKRGLLSDCEEQMVIDLHAQLGNRWS

BnMYB43-4 (1) MGRQPCCDKVGLKKGPWTAEEDKKLINFILTNGHCCWRALPKLSGLLRCGKSCRLRWINYLRPDLKRGLLSECEEQMVIDLHAQLGNRWS

BoMYB43-2 (1) MGRQPCCDKVGLKKGPWTAEEDKKLINFILTNGHCCWRALPKLSGLLRCGKSCRLRWINYLRPDLKRGLLSECEEQMVIDLHAQLGNRWS

Consensus (1) MGRQPCCDKVGLKKGPWTVEEDKKLINFILTNGHCCWRALPKLSGLLRCGKSCRLRWINYLRPDLKRGLLSE EEQMVIDLHAQLGNRWS

91 180

AtMYB43 (91) KIASHLPGRTDNEIKNHWNTHIKKKLRKMGIDPLTHKPLSEQEASQQ-AQGRKKSLVPHDDKNPKQ---DQQTKDEQ--EQHQLEQALEK

BnMYB43-1 (91) KIASHLPGRTDNEIKNHWNTHIKKKLRKMGIDPMTHKPLSEQDGAQQQAQGRKKSLVSRDEKNTNQGQEDQQQTRKEEDQQHQLGKDLEK

BrMYB43-1 (91) KIASHLPGRTDNEIKNHWNTHIKKKLRKMGIDPMTHKPLSEQDGAQQQAQGRKKSLVSRDEKNTNQGQEDQQQTRKEEDQQHQLGKDLEK

BnMYB43-2 (91) KIASHLPGRTDNEIKNHWNTHIKKKLRKMGIDPMTHKPLSEQDGAQQQAQGIKKSLVSRDEKNTNQGQEDQQQTRKEEDQQHQLGKDLEK

BoMYB43-1 (91) KIASHLPGRTDNEIKNHWNTHIKKKLRKMGIDPMTHKPLSEQEGAQQQAQGTKKSLVSRDEKKRNQGQ-EDQQTRKEEDQQHQLGKHLDK

BnMYB43-3 (91) KIASHLPGRTDNEIKNHWNTHIKKKLRKMGIDPMTHKPLSEQDGSQQ-AQG-SKSLVPHDDKNKKQDQEDKQTKEKQ--EHHQLGIDLDK

BrMYB43-2 (91) KIASHLPGRTDNEIKNHWNTHIKKKLRKMGIDPMTHKPLPEQDGSQQ-AQG-SKSLVPHDDKNKKQDQEDKQTKEKQ--EHHQLGIDLDK

BnMYB43-4 (91) KIASHLPGRTDNEIKNHWNTHIKKKLRKMGIDPMTHKPLPEQDGSQQ-AQG-SKSLVPHDDKNKKQDQEDKQTKEKQ--EHHQLGIDLDK

BoMYB43-2 (91) KIASHLPGRTDNEIKNHWNTHIKKKLRKMGIDPMTHKPLPEQDGSQQ-AQG-SKGLVPHDDKNKKQDQEDKQTKEKQ--EHHQLGIDLDK

Consensus (91) KIASHLPGRTDNEIKNHWNTHIKKKLRKMGIDPMTHKPLSEQDGSQQ AQG KKSLVPHDDKNKKQ QED QTKEKQ EQHQLG DLDK

181 270

AtMYB43 (175) NN-TSVSGDGFCIDEVPLLNPHEILIDISSSHHHHSNDDNVNINTSKFTSPSSSSSSTSSCISSVVPGDEFSKFFDEMEILDLKWLSSDD

BnMYB43-1 (181) NNTPISTDDGFCIDDIPLLSPHEIFVDIPSAHHHTS-DDNANINISNSTSPSSSSSSTSSCISSLP-GDEFSKFLDEMEIVDLKWLSSDD

BrMYB43-1 (181) NNTPISTDDGFCIDDIPLLSPHEIFVDISSAHHHTS-DDNANINISNSTSPSSSSSSISSCISSLP-GDEFSKFLDEMEIVDLKWLSSDD

BnMYB43-2 (181) NNTPISTDDGFCIDDIPLLSPHEIFVDISSAHHHTS-DDNANINISNSTSPSSSSSSTSSCISSLP-GDEFSKFLDEMEIVDLKWLSSDD

BoMYB43-1 (180) NNTPISTDDGFCIDDIPLLNPHEIFVDISSAHHHTS-DGNANINISNSTSPSSSSSPTSSCISSLP-GDEFSKFLDEMEIVDLKWLSSDD

BnMYB43-3 (177) SNKTISTDDGFCIDEVPLLDPHEILLDISFADHHTS-DENAHINISNSTSPSSSS--TSSCISSVP-GEEFSKFLDEMDTIDLKWLSSDH

BrMYB43-2 (177) SNKTISTDDGFCIDEVPLLDPHEILLDISFADHHTS-DENAHINISNSTSPSSSS--TSSCISSVP-GEEFSKFLDEMDTIDLKWLSSDH

BnMYB43-4 (177) SNKTISTDDGFCIDEVPLLDPHEILLDISFAQHHTS-DENAHINISNSTSPSSSS--TSSCISSVP-GEEFSKFLDEMETIDLKWLSSDH

BoMYB43-2 (177) SNKTISTDDGFCIDEVPLLDPHEILLDISFAQHHTS-DENAHINISNSASPSSSS--TSSCISSVP-GEEFSKFLDEMETIDLKWLSSDH

Consensus (181) NN TISTDDGFCIDEVPLL PHEILLDISSAHHHTS DDNANINISNSTSPSSSSS TSSCISSVP GDEFSKFLDEMEIIDLKWLSSDD

271 334

AtMYB43 (264) SLGDDISKDGKFNNSTVDTMNLWDINDLSSLDMFMNEHDDGFIGNGNGCSRMVLDQDSWTFDLL

BnMYB43-1 (269) SSGGNINYNDGKFNNNVDTMSLWDINDLSSLELFMNDHDDSVVEAD--VQEWF-----------

BrMYB43-1 (269) SSGGNINYNDGKFNNNVDTMSLWDINDLSSLELFMNDHDDSVVEAD--VQEWF-----------

BnMYB43-2 (269) SSGGDINYNDGKFNNNVDTMSLWDINDLSSLELFYE----------------------------

BoMYB43-1 (268) SSGGDINYKDGKFNNNVDTMNLWDINDLSSLELFMNDHDDSVVGSG--CSRMVLDHDSWTFDLL

BnMYB43-3 (263) SSEDNIIYKDDKFHNNVDTMNLWDINYLSSLELFMNDHDDGFVGSG--CSRMV-----------

BrMYB43-2 (263) SSEDNIIYKDDKFHNNVDTMNLWDINYLSSLELFMNDHDDGFVGSG--CSRMV-----------

BnMYB43-4 (263) SSGDNIIYKDDKFHNNVDTMNLWDINYLSSLELFMNDHDDGFVGSG--CSRMV-----------

BoMYB43-2 (263) SSGDNIIYKDDKFHNNVDTMNLWDINYLSSLELFMNDHDDGFVGSG--CSRMV-----------

Consensus (271) SSGDNI YKD KFNNNVDTMNLWDINDLSSLELFMNDHDDGFVGSG CSRMV

**(d)**


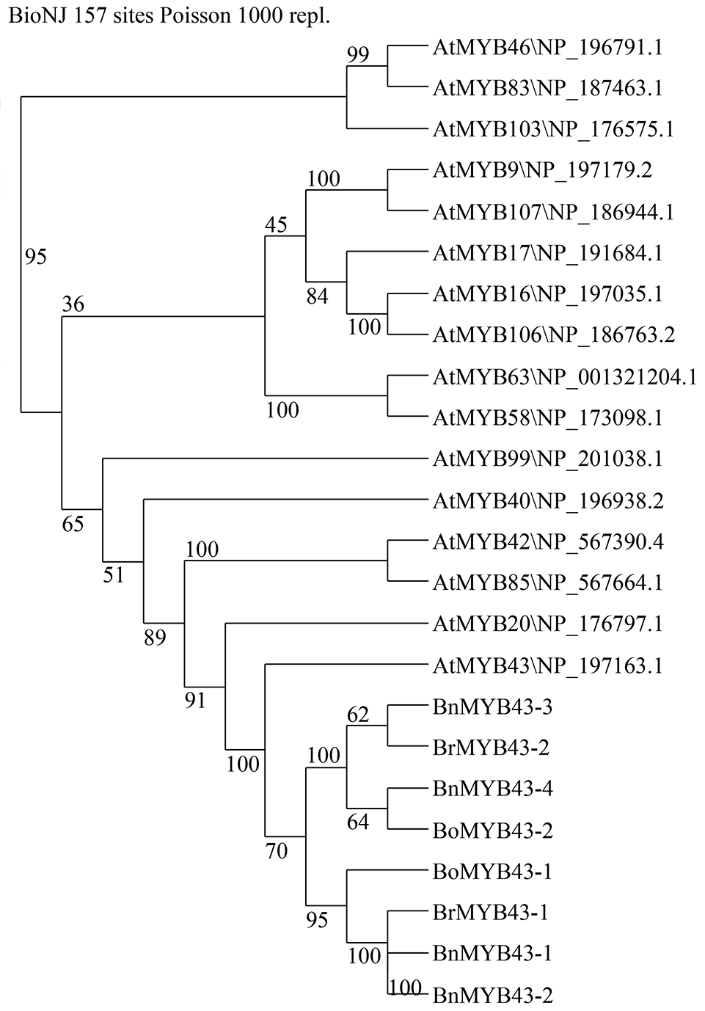


**Figure S4.** Nucleic acids and proteins analysis of the *MYB43* gene families from *B. napus*, *B. rapa*, *B.* *oleracea and A. thaliana*. (a) Sequence alignment of *BnMYB43*, *BrMYB43* and *BoMYB43* with *AtMYB43* (AT4G01680). (b) Gene structure of *BnMYB43*, *BrMYB43* and *BoMYB43* with *AtMYB43.* (c) Alignment of BnMYB43, BrMYB43 and BoMYB43 with AtMYB43 proteins. (d) Phylogenetic relationships of putative BnMYB43, BrMYB43 and BoMYB43 with AtMYB43 and other AtMYB proteins. The phylogenetic tree was generated using the SeaView4.0 program with the NJ method (1000 bootstrap replicates). At, *Arabidopsis thaliana*. Bn, *Brassica napus*. Br, *Brassica rapa*. Bo, *Brassica oleracea*.


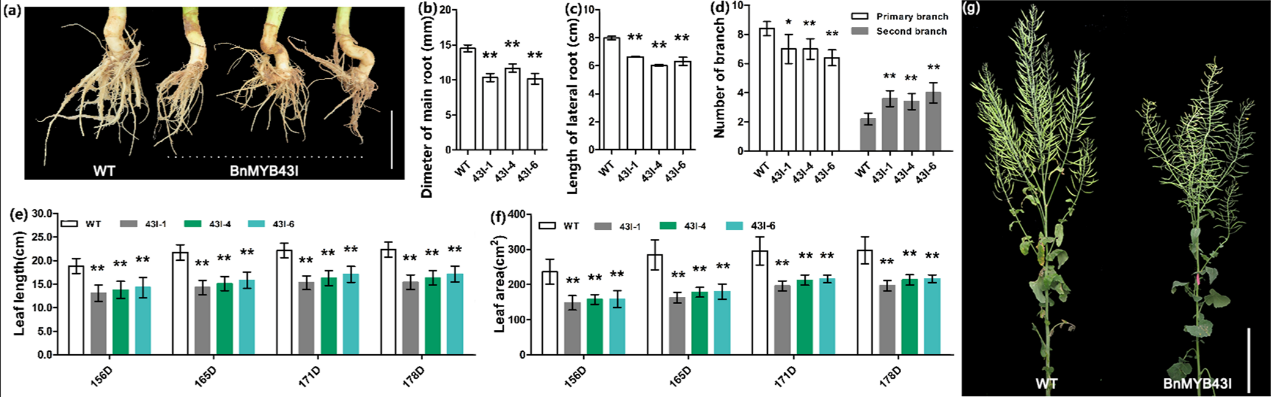


**Figure S5.** The plant phenotype of BnMYB43I transgenic plants. (a) The root phenotype of BnMYB43I transgenic plants. (b) The diameter of main root. (c) The length of lateral root in the bolting period. (d) The branch phenotype of BnMYB43I transgenic plants. (e, f) The development dynamic of the leaves’ length and area, respectively. The 14^th^ to 16^th^ leaves were signed for the survey. (g) The whole plant phenotype of BnMYB43I lines in the pod filling stage. Bar, 15 mm (a), 30 cm (g). Values are means ± SD. Asterisks indicate significant differences from the control (*, 0.01 ≤ P < 0.05; **, P < 0.01) using one-way ANOVA.
